# Streptococcal mutanobactin mediates interspecies competition with gram-positive bacteria

**DOI:** 10.1128/mbio.01623-26

**Published:** 2026-10-06

**Authors:** Ruth Y. Isenberg, Celine W. Sackih, Fatimah A. Abdulrahman, Julia L. E. Willett

**Affiliations:** 1 Department of Microbiology and Immunology, University of Minnesota Medical School 12269, Minneapolis, Minnesota, USA; University of Colorado Anschutz Medical Campus School of Medicine, Aurora, Colorado, USA

**Keywords:** *Streptococcus mutans*, *Enterococcus*, ESKAPE pathogens, VRE, mutanobactin, specialized metabolite, bacterial competition

## Abstract

Polymicrobial communities where bacteria must compete with each other to persist can serve as a source of uncharacterized antibacterial compounds to develop drugs for the treatment of drug-resistant infections. This study investigates interbacterial competition between bacteria found in the oral cavity, where *Streptococcus* species comprise a large portion of the resident oral microbiota, and *Enterococcus faecalis* is a pathobiont that is commonly found in root canal infections. We used co-cultures to determine whether oral streptococci and *E. faecalis* compete with each other. Our experiments revealed that multiple strains of *Streptococcus mutans*, an important cariogenic bacterium, reduce the viability of vancomycin-resistant *Enterococcus* and other gram-positive bacteria, including *Staphylococcus epidermidis* and methicillin-resistant *Staphylococcus aureus*. Furthermore, reduction in viability of bacteria by some strains of *S. mutans* requires the production of the non-ribosomal cyclic lipopeptide mutanobactin, while another strain inhibits independently of mutanobactin. We determined that *S. mutans* mutanobactin production increases target cell membrane permeability and that reduction of target cell viability is contact dependent. We also determined that the secreted *E. faecalis* protease gelatinase (GelE) is required for recovery from mutanobactin-mediated reduction in viability. Additionally, our data show that *S. mutans* mutanobactin production prevents *E. faecalis* biofilm formation and kills biofilm-associated *E. faecalis*. Together, this work demonstrates how natural products from a common oral bacterium contribute to competition in polymicrobial environments, which will inform future strategies to treat and prevent bacterial infections.

**IMPORTANCE:**

Antimicrobial resistance requires new therapeutics to treat drug-resistant infections. Novel antimicrobial compounds can be discovered in polymicrobial communities, where bacterial natural products promote competitive fitness. The oral cavity hosts a microbial consortium, and we investigated interactions between oral streptococci and *Enterococcus faecalis*, a common cause of root canal infections. We demonstrate antibacterial activity of streptococcal mutanobactin against gram-positive pathogens, including antibiotic-resistant isolates. We further show that the *E. faecalis* virulence factor gelatinase promotes recovery from mutanobactin-mediated reduction in viability, and that mutanobactin prevents and kills *E. faecalis* biofilms. By probing interactions between bacteria that occupy the same niche and characterizing antibacterial activity of a bacterial product, this work contributes to broader efforts to identify and develop antibiotics to treat clinically relevant drug-resistant infections.

## INTRODUCTION

Multi-drug-resistant bacterial infections pose a significant threat to public health and place a large financial burden on healthcare systems. The ESKAPE pathogens (*Enterococcus faecium*, *Staphylococcus aureus*, *Klebsiella pneumoniae*, *Acinetobacter baumannii*, *Pseudomonas aeruginosa*, and *Enterobacter* spp.) are responsible for a large portion of hospital-acquired antibiotic-resistant infections (1, 2). Additionally, vancomycin-resistant *Enterococcus* (VRE) is one of the most alarming antibiotic resistance threats identified by the U.S. Centers for Disease Control and Prevention and costs the United States over 4.6 billion dollars per year (3). *Enterococcus faecalis* and *Enterococcus faecium* are gram-positive gastrointestinal pathobionts that are leading causes of many hospital-acquired infections, such as endocarditis, bacteremia, urinary tract infections, and wound/surgical site infections (4–12). VRE isolated from patients harbor a number of genetic elements that promote virulence, including the enterococcal pathogenicity island (13–19), and resistance to last-resort antibiotics such as vancomycin can lead to drug-resistant strain dominance in the gastrointestinal tract that can promote bloodstream infection following antibiotic treatment (12, 20–23). While antibiotics such as linezolid, daptomycin, and tigecycline are used to treat VRE, resistance to these drugs is of increasing concern (5, 19, 21, 23). Therefore, the identification and development of new treatments to combat antimicrobial resistance are of critical importance.

While *E. faecalis* is a gastrointestinal commensal bacterium, it is also found in the oral cavity. It is frequently detected in the saliva and root canals of patients with apical periodontitis, most prominently as secondary or post-treatment infections (24–40) with a prevalence in secondary root canal infections of 24%–90% (40). It is hypothesized that *E. faecalis* is introduced to the oral cavity through a foodborne route, transiently persists in the oral cavity, and then establishes as a secondary infection in the root canal as a result of its ability to survive endodontic procedures and treatments (37, 40). *E. faecalis* is generally present in low abundance in the oral cavity; however, the presence of *E. faecalis* in saliva is associated with post-treatment apical periodontitis (25, 29). Furthermore, *E. faecalis* isolated from the saliva and root canal of the same patient has been found to be genetically related, suggesting that saliva may serve as a reservoir for *E. faecalis* root canal infections (29, 40). Many oral isolates of *E. faecalis* are resistant to antibiotics, suggesting that the oral cavity could also act as a reservoir for virulent and drug-resistant *E. faecalis* strains that cause hospital-acquired infections (35). Understanding *E. faecalis* mechanisms for survival within the oral cavity will broaden our knowledge of *E. faecalis* strategies for persistence and subsequent dissemination to infection sites.

The oral cavity is host to a diverse consortium of species, with the *Streptococcus* genus among the most abundant. Some *Streptococcus* species colonize specific and distinct oral sites, while others are found in all oral sites but reach higher abundance in specific sites (41, 42). For example, *Streptococcus mutans*, an oral pathogen linked to tooth decay and the development of caries (43), is abundant in supragingival plaque (41, 44). However, *S. mutans* has also been detected within infected root canals, possibly introduced via deep caries or contamination during endodontic procedures (45, 46). Because of the abundance and distribution of *Streptococcus* species within the oral cavity, the presence of *E. faecalis* in the oral cavity, and the prevalence of *E. faecalis* in infected root canals, we asked whether *Streptococcus* species compete with *E. faecalis* in co-culture. Through co-culture experiments, we revealed competitive interactions between multiple strains of *S. mutans* and various gram-positive pathogens. All together, our work characterizes interbacterial competition between *E. faecalis* and *S. mutans* as well as describes the antibacterial function for the previously identified natural product mutanobactin. Our findings highlight the utility in examining well-characterized microbial consortia for undiscovered mechanisms of interbacterial competition mediated by natural products. These compounds may be leveraged into new treatments for antibiotic-resistant infections caused by VRE and the ESKAPE pathogens.

## RESULTS

### *S. mutans* UA159 reduces the viability of gram-positive pathogens in co-culture

**Fig 1:**
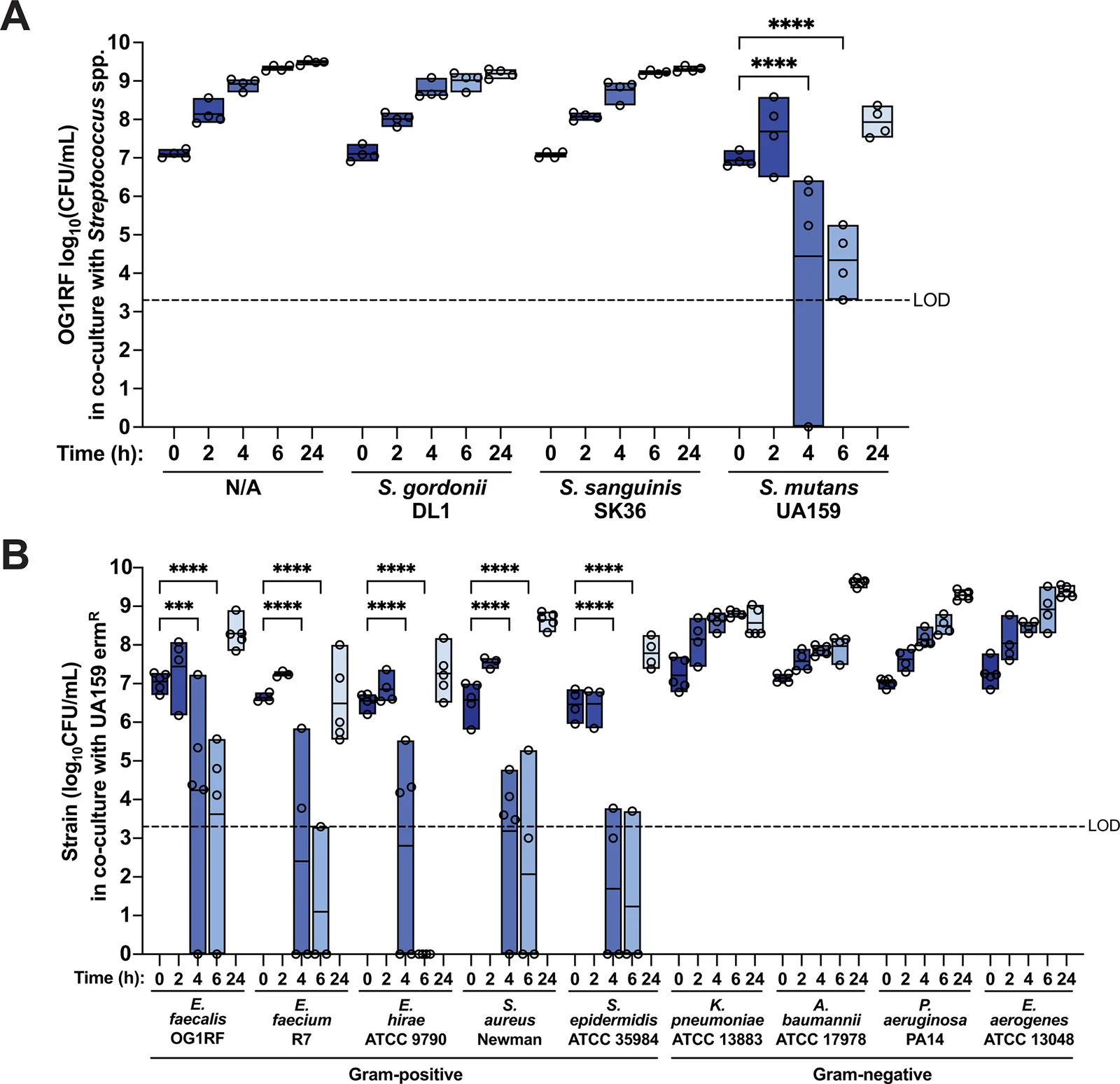
*S. mutans* UA159 reduces the viability of *E. faecalis* and other gram-positive pathogens in co-culture. (**A**) *E. faecalis* OG1RF CFU per milliliter in 1:10 co-culture with indicated *Streptococcus* spp. strain in BHI at 0, 2, 4, 6, and 24 h. For each culture, *n* = 2 technical replicates for each of *n* = 4 biological replicates. (**B**) CFU per milliliter of the indicated strains in 1:10 co-culture with *S. mutans* UA159 erm^R^ in BHI at 0, 2, 4, 6, and 24 h. For each culture, *n* = 2 technical replicates for each of *n* = 4–5 biological replicates. For panels **A** and **B**, two-way ANOVA was used for statistical analysis (***, *P* ≤ 0.001; ****, *P* ≤ 0.0001; not significant and significantly higher mean relative to 0 h comparisons are not shown); each dot represents the mean of technical replicates; floating bars represent the minimum, mean, and maximum; dashed lines indicate the limit of detection (LOD); and data points at *y* = 0 indicate no CFUs were detected.

*E. faecalis* is found in human oral cavities (25, 27, 34–36) and is commonly found in root canal infections (37, 38). Therefore, we asked whether *E. faecalis* and oral streptococci compete with each other. We co-cultured *Streptococcus gordonii* DL1, *Streptococcus sanguinis* SK36, and *S. mutans* UA159 (serotype c) with *E. faecalis* strain OG1RF, a derivative of a human oral isolate (27). We performed the co-cultures using a 1:10 *E. faecalis:Streptococcus* inoculum ratio in brain-heart infusion (BHI) broth and quantified CFU per milliliter of *E. faecalis* OG1RF at *t* = 0, 2, 4, 6, and 24 h. OG1RF CFU per milliliter increased over the course of 24 h when grown in BHI monoculture, and co-culture with *S. gordonii* and *S. sanguinis* did not affect OG1RF growth (Fig. 1A). However, OG1RF growth was significantly decreased by 2–3 logs compared to the inoculum of 10^7^ CFU/mL at 4 and 6 h of co-culture with *S. mutans* UA159, indicating that co-culture with UA159 results in loss of *E. faecalis* viability (Fig. 1A). This is consistent with a previous observation that UA159 inhibits *E. faecalis* on solid media (47). At 24 h of co-culture with UA159, we observed an increase in OG1RF CFU per milliliter to near inoculum levels and a 3.6 log increase compared to the 6 h time point (Fig. 1A), suggesting that the OG1RF population is able to recover from reduction in viability. We then performed co-cultures of OG1RF with UA159 at inoculation ratios between 1:1 and 1:10 and found that UA159 reduction of OG1RF viability is dose dependent (Fig. S1A). Conversely, UA159 growth increased during co-culture with OG1RF, with a slight decrease in CFU per milliliter at 24 h compared to 6 h (Fig. S1B). Because we observed the largest effect with a 1:10 ratio of OG1RF:UA159, we proceeded to use this ratio for subsequent co-culture experiments.

We then asked if *S. mutans* UA159 is able to reduce the viability of other strains of *E. faecalis* and performed co-culture experiments with 11 other *E. faecalis* clinical isolates (listed in Table S1). To facilitate selection of UA159 for CFU enumeration, we constructed a strain of UA159 with an erythromycin resistance cassette integrated at a neutral chromosomal site (UA159 erm^R^) and used this strain for all subsequent co-culture experiments. When co-cultured with UA159, all 11 *E. faecalis* strains tested had lower CFU per milliliter at 4 h compared to 0 h, including the vancomycin-resistant strain V583 (Fig. S2A). None of the *E. faecalis* isolates tested had growth defects in BHI monoculture performed in parallel with the co-cultures (Fig. S2B). We then expanded our panel to include *Enterococcus faecium* and *Enterococcus hirae* isolates. Again, when co-cultured with UA159, viability of all *Enterococcus* isolates was reduced at 4 h compared to 0 h (Fig. 1B; Fig. S2C), and none of the *Enterococcus* isolates tested had growth defects in BHI monocultures performed in parallel (Fig. S1D). We further expanded our panel to include both gram-positive and gram-negative pathogens, with a representative isolate for each ESKAPE pathogen. In addition to the *Enterococcus* spp. strains tested, the viability of *S. aureus* and *S. epidermidis* was also reduced by multiple orders of magnitude by UA159 at 4 h of co-culture (Fig. 1B), with many cultures reaching the lower limit of detection by 6 h of co-culture with UA159. The growth of the gram-negative pathogens (*Klebsiella pneumoniae*, *Acinetobacter baumannii, Pseudomonas aeruginosa*, and *Enterobacter aerogenes*) was not impacted by co-culture with UA159 (Fig. 1B). Altogether, these data suggest that the *S. mutans* UA159 target range includes gram-positive ESKAPE pathogens but does not extend to gram-negative bacteria.

### *E. faecalis* gelatinase activity promotes recovery from reduction of viability by *S. mutans* UA159

At 24 h of co-culture with *S. mutans* UA159, we observed OG1RF recovery from reduction in viability, and 24 h CFU per milliliter values were similar to the inoculum (Fig. 1A). Because the CFU per milliliter of UA159 in co-culture with OG1RF increased over 24 h (Fig. S1B), the multi-log increase of OG1RF CFU per milliliter at 24 h was not due to fewer UA159 cells in co-culture. To test whether OG1RF acquires lasting resistance to reduction in viability by UA159, we isolated OG1RF colonies from a 24 h co-culture with UA159 and reinoculated them into a new co-culture with UA159. The viability of the 24 h co-culture isolates was reduced by UA159 to the same degree as the OG1RF parent strain (Fig. S3A), suggesting that OG1RF recovery is not the result of any mutations that may have occurred during co-culture and instead may be due to an OG1RF factor that promotes recovery.

**Fig 2:**
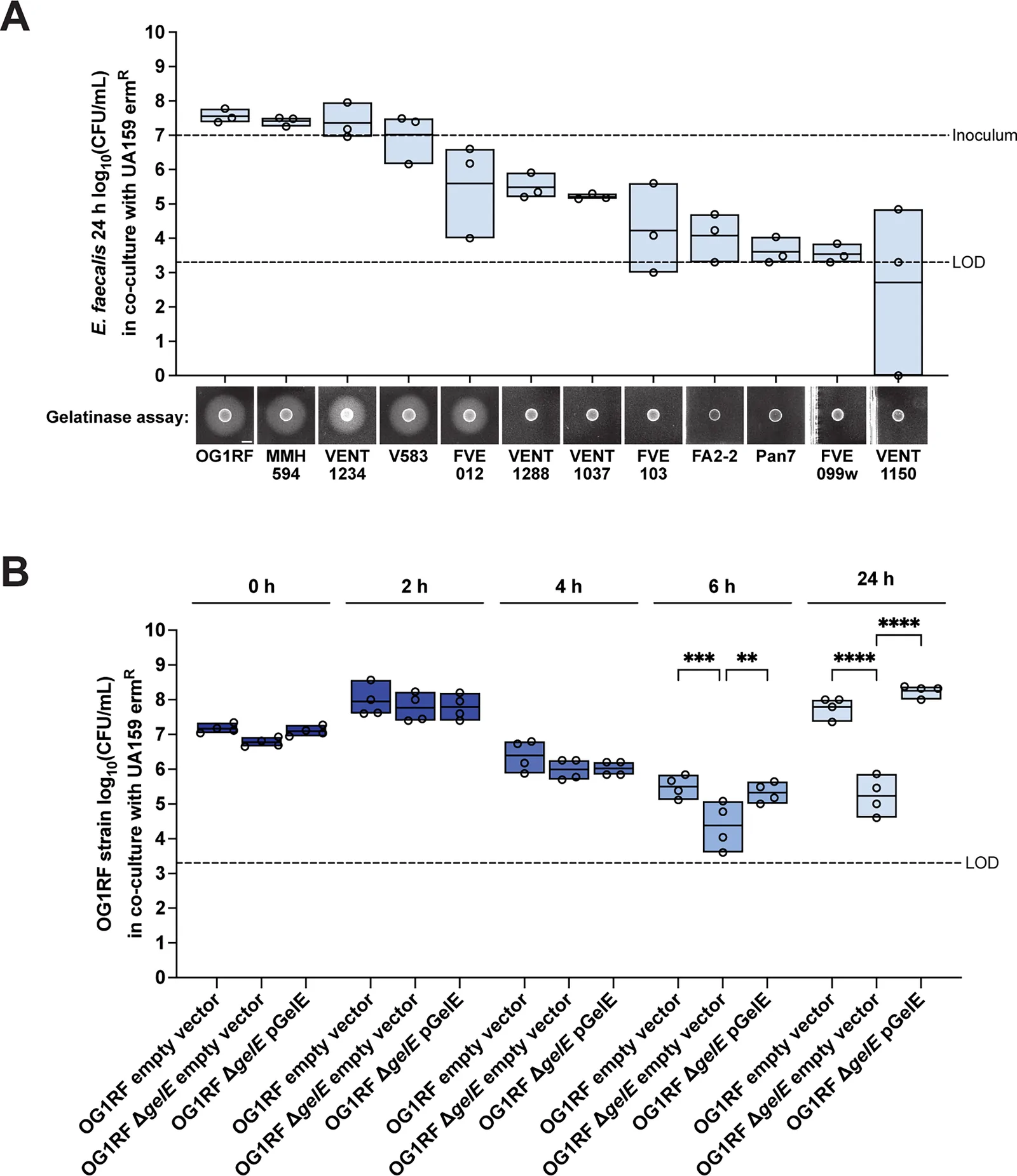
*E. faecalis* gelatinase GelE is required for recovery from reduction in viability by *S. mutans* UA159. (**A**) CFU per milliliter of the indicated *E. faecalis* clinical isolates in 1:10 co-culture with *S. mutans* UA159 erm^R^ in BHI at 24 h (data are the same as shown in Fig. S2A). Representative gelatinase assay images are included on the *x*-axis for each *E. faecalis* clinical isolate. For each co-culture, *n* = 2 technical replicates for each of *n* = 3 biological replicates. For the gelatinase assays, *n* = 2 technical replicates per *n* = 2–3 biological replicates. Upper dashed lines indicate the *E. faecalis* inoculum of 10^7^ CFU/mL; lower dashed lines indicate the limit of detection (LOD). Data points at *y* = 0 indicate no CFUs were detected. The scale bar represents 5 mm. (**B**) CFU per milliliter of the indicated OG1RF strains in 1:10 co-culture with *S. mutans* UA159 erm^R^ in BHI at 0, 2, 4, 6, and 24 h. For each culture, *n* = 2 technical replicates for each of *n* = 4 biological replicates. Two-way ANOVA was used for statistical analysis (**, *P* ≤ 0.01; ***, *P* ≤ 0.001; ****, *P* ≤ 0.0001; not significant comparisons are not shown). Dashed line indicates the LOD. For panels A and B, each dot represents the mean of technical replicates; floating bars represent the minimum, mean, and maximum.

While testing the panel of *E. faecalis* isolates shown in Fig. S2A, we noticed that several isolates, in addition to OG1RF, recovered from reduction in viability by 24 h of co-culture, but some did not recover by 24 h. One of the *E. faecalis* strains that did not recover is FA2-2 (Fig. 2A), which has a mutation in the *fsr* quorum sensing system that controls the expression of GelE, an extracellular protease that is a key virulence factor for *E. faecalis* (48–51). Conversely, strains known to have gelatinase activity, OG1RF and V583 (49, 52), did recover from reduction in viability (Fig. 2A). This led us to hypothesize that gelatinase activity of *E. faecalis* predicts recovery from reduction in viability by UA159. To determine the gelatinase activity of the *E. faecalis* strains we tested in co-culture with UA159, we used a gelatin plate-based assay to detect gelatinase activity after overnight growth. Of the 12 *E. faecalis* strains assayed, five displayed gelatinase activity as indicated by an opaque halo around the site where strains were spotted (Fig. 2A). Four of the gelatinase-positive strains, OG1RF, MMH594, VENT1234, and V583, recovered from reduction in viability at or above the level of inoculum, while no gelatinase-negative strains recovered (Fig. 2A). FVE012 is gelatinase positive, and although it did not recover to the level of inoculum, it did recover 1–3 logs more than a majority of the gelatinase-negative strains (Fig. 2A). We quantified gelatinase activity by measuring the radius of the opaque halo surrounding the strains spotted on gelatin plates and found that FVE012 had reduced gelatinase activity compared to most of the other gelatinase-positive strains (Fig. S3B). Therefore, decreased gelatinase expression may explain the intermediate recovery of FVE012 from reduction in viability. Indeed, when we plotted average 24 h co-culture CFU per milliliter and average gelatinase halo radius for each of the *E. faecalis* strains, strains with any gelatinase activity recovered from killing more than strains without gelatinase activity (Fig. S3B). Interestingly, VENT 1234 had less gelatinase activity than FVE012 yet recovered as well as the strains with higher gelatinase activity (Fig. S3B). This may indicate that additional factors contribute to *E. faecalis* recovery. Together, our gelatinase assay and co-culture results suggest that *E. faecalis* gelatinase activity predicts recovery from reduction in viability by UA159. This observation is also consistent with the timing of GelE activity reaching its peak when growth reaches the stationary phase (53).

To determine the requirement of gelatinase for recovery from killing by UA159, we co-cultured a strain of OG1RF lacking the GelE gelatinase with UA159. While the OG1RF Δ*gelE* strain did not have a growth defect in monoculture (Fig. S3C), OG1RF Δ*gelE* had significantly fewer CFU per milliliter at 24 h of co-culture than the OG1RF parental strain, and expression of *gelE* from an inducible plasmid restored the OG1RF Δ*gelE* recovery defect (Fig. 2B). These results demonstrate that gelatinase activity is required for full *E. faecalis* recovery from reduction in viability by UA159.

We next asked whether extracellular proteases could protect other gram-positive bacteria from reduction in viability by UA159 since the *S. aureus* strains Newman, RN4220, and LAC* recovered from killing by 24 h (Fig. 1B; Fig. S3D and E). *S. aureus* produces several extracellular proteases (54, 55), so we wondered if any of these enzymes contribute to recovery from killing by UA159. We performed co-cultures with UA159 and the *S. aureus* MRSA strain LAC* lacking four known extracellular proteases (Aur, SspAB, ScpA, and Spl) and found that the LAC* protease knockout strain recovered from killing similarly to the parental LAC* strain (Fig. S3E). This result demonstrates that *S. aureus* recovery from killing by UA159 is not mediated by the production of these extracellular proteases and that mechanisms of killing and/or recovery from killing may vary depending on the target species.

### *S. mutans* ATCC 33535 reduces the viability of *E. faecalis* and other gram-positive pathogens

**Fig 3:**
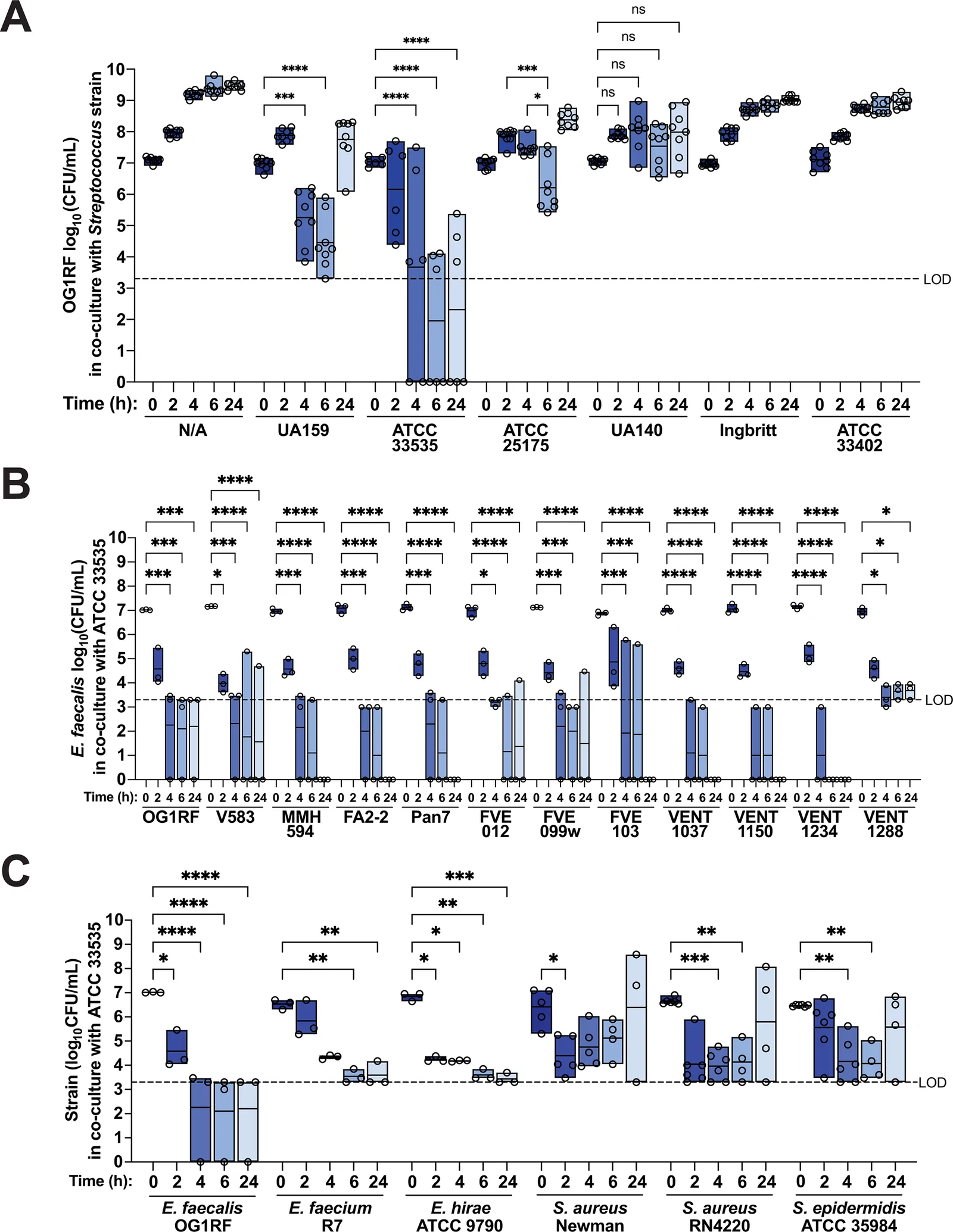
*S. mutans* ATCC 33535 reduces the viability of *E. faecalis* and other gram-positive pathogens in co-culture. (**A**) *E. faecalis* OG1RF CFU per milliliter in 1:10 co-culture with the indicated *S. mutans* strain or *S. sobrinus* ATCC 33402 in BHI at 0, 2, 4, 6, and 24 h. For each culture, *n* = 2 technical replicates for each of *n* = 6–8 biological replicates. (**B**) CFU per milliliter of the indicated *E. faecalis* clinical isolates in 1:10 co-culture with *S. mutans* ATCC 33535 in BHI at 0, 2, 4, 6, and 24 h. For each culture, *n* = 2 technical replicates for each of *n* = 3 biological replicates. (**C**). CFU per milliliter of the indicated strains in 1:10 co-culture with *S. mutans* ATCC 33535 in BHI at 0, 2, 4, 6, and 24 h. For each culture, *n* = 2 technical replicates for each of *n* = 3–6 biological replicates. For panels **A–C**, two-way ANOVA was used for statistical analysis (ns, not significant; *, *P* ≤ 0.05; **, *P* ≤ 0.01; ***, *P* ≤ 0.001; ****, *P* ≤ 0.0001); for panel A, only comparisons to the 0 h time point are shown except for ATCC 25175; ns comparisons are not shown except for UA140; for panels **B** and **C**, only comparisons to the 0 h time point are shown; ns comparisons are not shown; and for panels **A–C**, significantly higher mean values relative to 0 h comparisons are not shown. Each dot represents the mean of technical replicates; floating bars represent the minimum, mean, and maximum; dashed lines indicate the limit of detection (LOD); and data points at *y* = 0 indicate no CFUs were detected.

Next, we investigated whether reduction of *E. faecalis* viability is specific to *S. mutans* UA159 or whether other strains of *S. mutans* have inhibitory activity. We performed co-culture experiments with *E. faecalis* OG1RF and four additional serotype c strains of *S. mutans*: UA140, ATCC 25174, ATCC 33535, and Ingbritt. We also included *Streptococcus sobrinus* strain ATCC 33402 (serotype g), which was previously classified as an *S. mutans* strain. Of the six *Streptococcus* strains tested, UA159, ATCC 25175, and ATCC 33535 significantly reduced CFU per milliliter of OG1RF (Fig. 3A). UA140 did not reduce viable OG1RF cells below that of the inoculum, but OG1RF growth was inhibited and did not significantly increase over time (Fig. 3A). We observed differences in the timing and magnitude of killing by UA159, ATCC 25175, and ATCC 33535. While UA159 reduced OG1RF CFU per milliliter starting at 4 h and by a maximum of 3.4 logs at 6 h compared to the highest CFU per milliliter at 2 h, ATCC 25175 only reduced OG1RF CFU per milliliter by 1.6 logs at 6 h compared to 2 h (Fig. 3A). Co-culture with ATCC 33535, however, reduced OG1RF CFU per milliliter as early as 2 h and reduced CFU per milliliter by a maximum of 5.1 logs at 6 h compared to 0 h (Fig. 3A). By 24 h of co-culture with ATCC 33535, OG1RF had not recovered from killing (Fig. 3A). Additionally, co-culture with ATCC 33535 eliminated any observable OG1RF CFUs for half of the biological replicates at 4, 6, and 24 h (Fig. 3A). Therefore, while multiple strains of *S. mutans* reduce OG1RF viability, there are notable differences among *S. mutans* strains in the timing and magnitude of these effects. This suggests strain-to-strain differences in the mechanisms of *S. mutans* targeting and *E. faecalis* recovery.

Because ATCC 33535 exhibited the strongest reduction of *E. faecalis* viability of all the *Streptococcus* strains tested, we proceeded to test it against the panel of strains we tested against UA159. Similarly to UA159, ATCC 33535 reduced the viability of all *E. faecalis* clinical isolates tested (Fig. 3B), while monocultures of each *E. faecalis* isolate performed in parallel increased in CFU per milliliter from 0 to 24 h (Fig. S4A). Interestingly, the 12 *E. faecalis* isolates did not recover from reduction in viability by ATCC 33535 at 24 h (Fig. 3A; Fig. S4B) as they did when co-cultured with UA159 (Fig. 2A). This failure of OG1RF viability to recover from reduction during co-culture with ATCC 33535, combined with our previous observation that ATCC 33535 reduces OG1RF viability earlier and to a larger magnitude than UA159, suggests the mechanism used by ATCC 33535 to reduce the viability of target cells differs from that used by UA159. ATCC 33535 also reduced *E. faecium*, *E. hirae*, *S. epidermidis*, and *S. aureus* viability (Fig. 3C), while monocultures of each of these strains performed in parallel increased in CFU per milliliter from 0 to 24 h (Fig. S4C). ATCC 33535 did not negatively impact the growth of any of the gram-negative bacteria tested (Fig. S4D). Therefore, while ATCC 33535 reduces target cell viability earlier and to a larger magnitude than UA159, ATCC 33535 may have the same target range as UA159.

### *S. mutans* UA159 mutanobactin biosynthesis genes are required to reduce the viability of target strains

**Fig 4:**
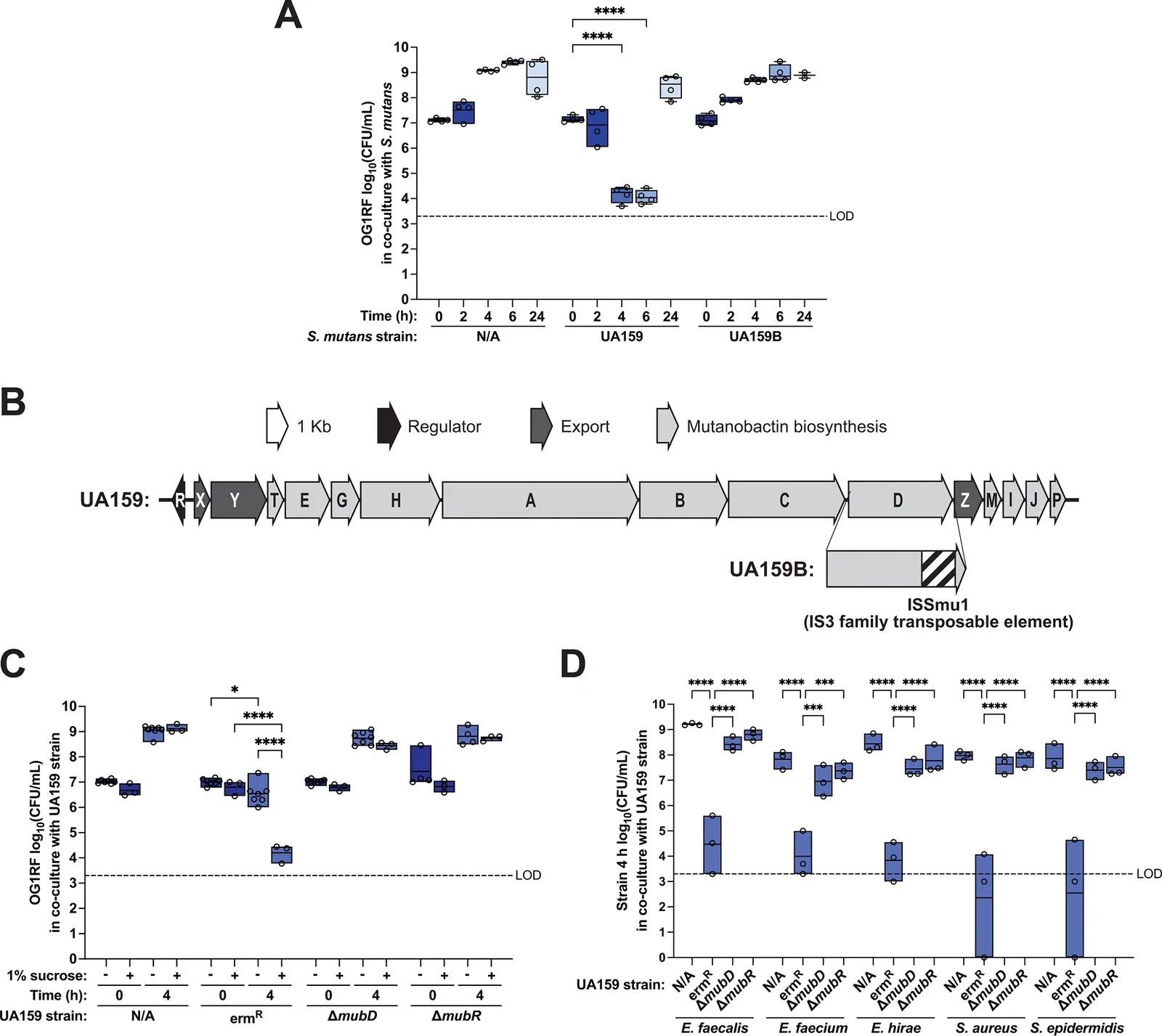
*S. mutans* UA159 mutanobactin genes are required to reduce the viability of target strains in co-culture. (**A**) *E. faecalis* OG1RF CFU per milliliter in 1:10 co-culture with the indicated *S. mutans* strain in BHI at 0, 2, 4, 6, and 24 h. For each culture, *n* = 2 technical replicates for each of *n* = 2–4 biological replicates. (**B**) Genetic organization of the *S. mutans* UA159 mutanobactin biosynthetic gene cluster and location of the UA159B ISSmu1 insertion in *mubD*. (**C**) *E. faecalis* OG1RF CFU per milliliter in 1:10 co-culture with the indicated *S. mutans* UA159 strain in BHI ± 1% sucrose at 0 and 4 h. For each culture, *n* = 2 technical replicates for each of *n* = 3–7 biological replicates. (**D**) CFU per milliliter of the indicated strains in 1:10 co-culture with the indicated *S. mutans* UA159 strain in BHI at 4 h. For each culture, *n* = 2 technical replicates for each of *n* = 3 biological replicates. For panels A, C, and D, two-way ANOVA was used for statistical analysis (*, *P* ≤ 0.05; ***, *P* ≤ 0.001; ****, *P* ≤ 0.0001); for panels A, C, and D, non-significant comparisons are not shown; for panels A and C, significantly higher mean values relative to 0 h comparisons are not shown. Each dot represents the mean of technical replicates; floating bars represent the minimum, mean, and maximum; dashed lines indicate the limit of detection (LOD); and data points at *y* = 0 indicate no CFUs were detected.

While testing different strains of *S. mutan*s, we obtained a separate stock of UA159 (referred to as UA159B). Interestingly, UA159B did not reduce the viability of OG1RF in co-culture, and growth of OG1RF in co-culture with UA159B was similar to that of OG1RF in monoculture (Fig. 4A). To determine whether a genetic difference could explain the inability of UA159B to reduce OG1RF viability in co-culture, we sequenced the genome of UA159B and compared it to the original isolate of UA159. This revealed an insertion mutation in UA159B *mubD* by ISSmu1, an IS3 family transposable element (Fig. 4B). The *mubD* gene is a part of the biosynthetic gene cluster that produces mutanobactin, a hybrid polyketide/non-ribosomal peptide (56, 57) that promotes oxygen and hydrogen peroxide stress tolerance (57–59), represses the yeast-mycelium morphological transition of the oral fungal pathogen *Candida albicans* (56, 60), and increases immunomodulatory activity (58). While synthesized mutanobactin has been shown to have some inhibitory activity against several oral bacterial species after 64 h of growth (61), mutanobactin production by *S. mutans* has not been shown to have antibacterial activity against enterococci or any of the other gram-positive pathogenic bacteria we tested. Therefore, we next aimed to determine whether *S. mutans* mutanobactin genes are required to reduce target cell viability.

To confirm that the mutation in *mubD* is responsible for the inability of UA159B to reduce the viability of OG1RF, we deleted *mubD* in our original UA159 strain background and co-cultured the Δ*mubD* strain with OG1RF. UA159 Δ*mubD* did not reduce OG1RF viability in co-culture (Fig. 4C; Fig. S5A), confirming that *mubD* is required for UA159 antibacterial activity. We also generated a deletion of *mubR*, which encodes a TetR family transcription factor that activates expression of the *mub* gene cluster (57). This UA159 Δ*mubR* mutant was also unable to reduce OG1RF viability in co-culture (Fig. 4C; Fig. S5A). The UA159 Δ*mubD* and Δ*mubR* mutants failed to reduce the viability of other gram-positive species tested as well (Fig. 4D). These results confirm that an intact mutanobactin biosynthetic gene cluster is required for UA159 to reduce target cell viability.

We next wanted to rule out other known aspects of *S. mutans* UA159 biology that could contribute to reduction of target strain CFU per milliliter during co-culture. We first tested whether UA159 altered culture pH such that co-culture strains were not able to survive, as *S. mutans* and other lactic acid bacteria (like *Enterococcus* spp.) reduce pH during growth (62). We measured the pH of 24 h monocultures of *E. faecalis* OG1RF and UA159 as well as an OG1RF/UA159 co-culture. Compared to uninoculated BHI, OG1RF decreased the culture pH to 5.3, UA159 decreased the culture pH to 5.0, and the OG1RF/UA159 co-culture pH matched that of a UA159 monoculture (Fig. S6A). We grew OG1RF in BHI at a range of pH values from 2 to 7 and determined that OG1RF viability was not reduced above pH 4 (Fig. S6B). Therefore, it is unlikely that acid production by UA159 is responsible for the reduction of OG1RF viability. *S. mutans* UA159 produces two non-lantibiotic mutacins, mutacin IV and mutacin V, encoded by *nlmAB* and *nlmC*, respectively (63). Since UA159 production of mutacin IV and V inhibits the growth of various *Streptococcus* spp. (64), we next tested whether mutacin production by UA159 is responsible for reducing OG1RF viability. When we co-cultured OG1RF with UA159 lacking genes for both mutacins, OG1RF growth was not different from OG1RF co-cultured with UA159 erm^R^ (Fig. S6C). Therefore, mutacin production by UA159 is not responsible for reducing OG1RF viability, and mutanobactin production is likely the primary pathway through which UA159 reduces target cell viability.

The mutanobactin gene cluster is encoded by many, but not all, *S. mutans* isolates (56). In addition to UA159, UA140 encodes genes for mutanobactin production (57). Therefore, we hypothesized that reduction of target cell viability by *S. mutans* is dependent on the mutanobactin gene cluster. To test this, we analyzed the genomes of each of the strains we co-cultured with *E. faecalis* (UA159, UA140, ATCC 25175, ATCC 33402, ATCC 33535, and Ingbritt). Analysis of these genomes revealed that the strains that did not reduce *E. faecalis* viability (Ingbritt and ATCC 33402) do not encode mutanobactin genes (Table 1). Conversely, we identified a putative mutanobactin biosynthetic gene cluster in the genome of ATCC 25175, which did reduce *E. faecalis* viability (Table 1). However, ATCC 33535, the strain with the largest impact on *E. faecalis* growth, does not encode genes for the production of mutanobactin (Table 1). To determine whether ATCC 33535 encodes other secondary metabolite regions that could produce natural products that contribute to reduction in *E. faecalis* viability, we analyzed the ATCC 33535 genomes using antiSMASH (65). This analysis revealed that the ATCC 33535 genome contains seven different regions predicted to encode biosynthetic gene clusters: four unspecified ribosomally synthesized and post-translationally modified peptide product (RiPP-like) clusters, one type III polyketide synthase cluster, one non-ribosomal peptide synthase (NRPS) cluster, and one hybrid highly reducing type II polyketide synthase-like ishigamide and skyllamycin/NRPS/type I polyketide synthase cluster (Table 2). None of the secondary metabolite regions predicted by antiSMASH had similarity to any known clusters. Based on these genomic analyes, we conclude that *S. mutans* UA159, UA140, and ATCC 25175 reduce target cell viability likely through production of mutanobactin. ATCC 33535 instead uses a mechanism independent of mutanobactin, and we hypothesize that one or more of the predicted secondary metabolite clusters encoded by ATCC 33535 contribute to reduction of target cell viability.

**TABLE 1:** *S. mutans* strains mutanobactin genotypes and phenotypes in co-culture with *E. faecalis*

*S. mutans* strain	Mutanobactin biosynthetic gene cluster?	Impacts *E. faecalis* growth?
UA159	Yes;,SMU.1334/SMU_1334 to SMU.1348c/SMU_1348c (NC_004350.2)	Yes
ATCC 33535	No	Yes
ATCC 25175	Yes; D820_06038 to D820_06163 (NZ_AOCB01000008.1)	Yes
UA140	Yes; *mubK-mubP* (57)	Yes
Ingbritt	No	No

**TABLE 2:** *S. mutans* ATCC 33535 secondary metabolite regions predicted by antiSMASH

Region	Cluster type^*a*^	Genomic location (nt)
Region 1	RiPP-like	236,957 to 248,315
Region 2	RiPP-like	703,132 to 714,169
Region 3	T3PKS	870,114 to 911,289
Region 4	NRPS	1,186,764 to 1,237,123
Region 5	RiPP-like	1,546,987 to 1,557,217
Region 6	RiPP-like	1,671,080 to 1,698,234
Region 7	HR-T2PKS, NRPS, T1PKS	1,797,066 to 1,861,346

^*a*^ NRPS, non-ribosomal peptide synthase; NR-T2PKS, hybrid highly reducing type II polyketide synthase like ishigamide and skyllamycin; RiPP-like, unspecified ribosomally synthesized and post-translationally modified peptide product; T1PKS, type I polyketide synthase; T3PKS, type III polyketide synthase.

We next aimed to determine whether *S. mutans* mutanobactin production mediates interbacterial competition in conditions relevant to the oral cavity. Sucrose promotes the formation of dental caries by lowering pH and serving as a substrate for extracellular and intracellular polysaccharide synthesis (62, 66–71). To see whether reduction in target cell viability by UA159 occurred in the presence of this physiologically relevant sugar, we performed co-culture assays between UA159 and *E. faecalis* OG1RF in BHI supplemented with 1% sucrose, which has been shown to reduce mutanobactin gene expression (72). At 4 h of co-culture with UA159, however, we observed enhanced reduction of OG1RF CFU per milliliter in BHI + 1% sucrose compared to unsupplemented BHI (Fig. 4C). Co-culture with either the UA159 Δ*mubD* or Δ*mubR* mutant in BHI + 1% sucrose did not reduce OG1RF CFU per milliliter compared to OG1RF monoculture (Fig. 4C; Fig. S5B). Both *mubD* and *mubR* were also required for UA159 to reduce viability of each of the other gram-positive species tested (Fig. 4D; Fig. S7A through E). These results indicate that mutanobactin genes are required to reduce target viability in the presence of sucrose.

In monoculture, *S. mutans* UA159 mutanobactin gene expression increases during exponential growth and peaks before dropping once the culture reaches stationary phase (57). We were interested in whether the presence of a target strain alters mutanobactin gene expression, or whether mutanobactin gene expression is unaffected in co-culture. To test this, we constructed two *mub* transcriptional reporter plasmids with GFP gene expression under the control of either the *mubR* promoter or the *mubX* promoter. We then used these reporter plasmids to measure *mub* expression by UA159 in mono- and co-culture with *E. faecalis* OG1RF. Activity of the *mubR* and *mubX* promoters was not significantly different when UA159 was cultured alone vs in co-culture with OG1RF (Fig. S7F), indicating that *mub* expression is not impacted by the presence of OG1RF. Taken all together, our results provide strong genetic evidence that *S. mutans* UA159 reduction of target cell viability is dependent on mutanobactin.

### *S. mutans* increases target cell membrane permeability

**Fig 5:**
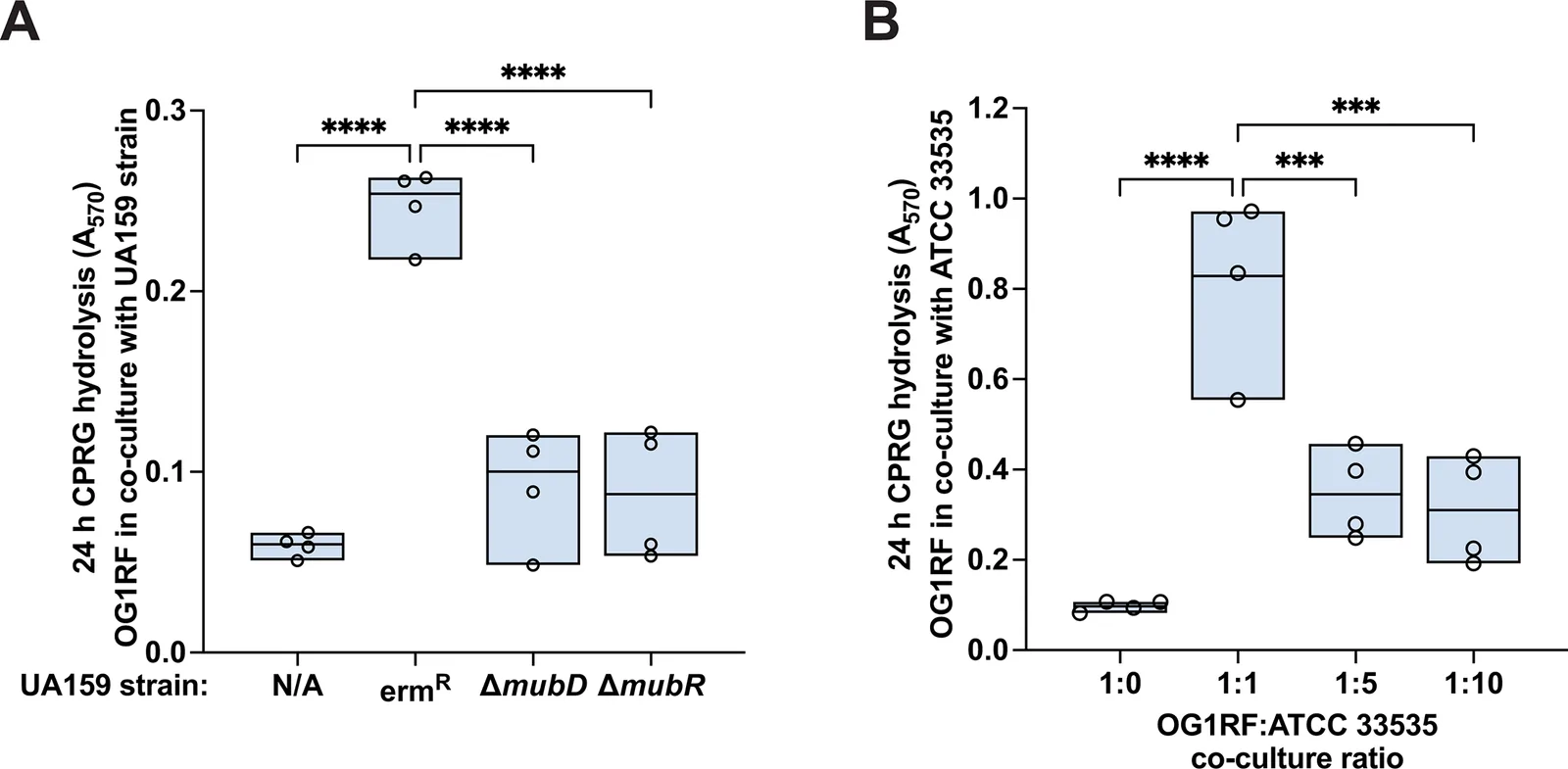
*S. mutans* UA159 and ATCC 33535 increase *E. faecalis* cell membrane permeability. (**A**) CPRG hydrolysis of *E. faecalis* OG1RF at 24 h co-culture with the indicated *S. mutans* UA159 strain in TBS-D. For each culture, *n* = 2 technical replicates for each of *n* = 4 biological replicates. (**B**) CPRG hydrolysis of *E. faecalis* OG1RF in 24 h co-culture with *S. mutans* ATCC 33535 at an indicated inoculum ratio of ATCC 33535 to 1 part OG1RF in TBS-D. For each culture, *n* = 2 technical replicates for each of *n* = 4 biological replicates. For panels A and B, one-way ANOVA was used for statistical analysis (***, *P* ≤ 0.001; ****, *P* ≤ 0.0001; non-significant comparisons are not shown). Each dot represents the mean of technical replicates; floating bars represent the minimum, mean, and maximum.

Mutanobactin is a cyclic lipopeptide that is similar in structure to daptomycin, a lipopeptide antibiotic that targets gram-positive bacterial cell membranes (56, 57, 60, 73). Daptomycin exerts its antibacterial effect by inserting into the gram-positive bacterial cell membrane, resulting in membrane depolarization (74, 75). Since mutanobactin and daptomycin share structural similarities, we hypothesized that mutanobactin also reduces target cell viability by targeting the bacterial cell membrane. To test this, we performed cell membrane permeability assays using chlorophenyl red-β-D-galactopyranoside (CPRG) to measure *E. faecalis* OG1RF cell membrane integrity (76, 77). In this assay, the β-galactosidase activity of LacZ constitutively expressed by OG1RF is assessed by measuring the absorbance of chlorophenyl red, the cleavage product of the cell membrane-impermeable compound CPRG. We cultured OG1RF constitutively expressing *lacZ* from the pCJK205 plasmid in BHI with subinhibitory concentrations of daptomycin and ciprofloxacin, a fluoroquinolone antibiotic that functions by inhibiting DNA replication (78). As expected, OG1RF cell membrane permeability relative to the untreated control was significantly increased when grown with daptomycin but not with ciprofloxacin (Fig. S8A). When we co-cultured OG1RF pCJK205 with *S. mutans* UA159, OG1RF cell membrane permeability was significantly higher than when grown in monoculture, and this increase was dependent on UA159 *mubD* and *mubR* (Fig. 5A). These results support our hypothesis that *S. mutans* UA159 production of mutanobactin reduces target cell viability by disrupting cell membrane integrity.

We also tested the ability of *S. mutans* ATCC 33535, which does not encode a biosynthetic gene cluster for mutanobactin production, to increase OG1RF cell membrane permeability. We observed a significant increase in OG1RF cell membrane permeability when we inoculated OG1RF pCJK205 and ATCC 33535 at a 1:1 ratio in the CPRG assay but not when ATCC 33535 was inoculated at higher ratios (Fig. 5B). We hypothesize that higher ratios of ATCC 33535 to OG1RF did not yield higher CPRG cleavage because at the higher ratios, ATCC 33535 disrupted the OG1RF cells to the point where not enough LacZ was expressed to produce the CRPG cleavage product. This is consistent with our co-culture data showing that ATCC 33535 reduces OG1RF viability faster and to a greater magnitude than UA159 (Fig. 3A). We confirmed that OG1RF without the LacZ plasmid does not significantly contribute to CPRG cleavage, nor does UA159 or ATCC 33535 (Fig. S8A).

We next tested whether *S. mutans* UA159 reduction of target cell viability is contact dependent by growing *E. faecalis* OG1RF in UA159 spent media. There was no difference in OG1RF growth in spent media derived from OG1RF or UA159 monocultures or from a 4 h 1:10 co-culture of OG1RF and UA159 (Fig. S8B), although our transcriptional reporter data confirmed that *mub* genes were expressed in monoculture and co-culture (Fig. S7F). Thus, mutanobactin-mediated antibacterial activity by UA159 is likely contact dependent. Interestingly, when we grew OG1RF in spent media from ATCC 33535, we observed a 1.7 log inhibition of OG1RF CFU per milliliter and similar inhibition when OG1RF was grown in spent media from OG1RF/ATCC 33535 co-cultures (Fig. S8B). While OG1RF viability in ATCC 33535 spent media was not reduced to the same extent as when co-cultured with ATCC 33535 cells (Fig. 3A; Fig. S8B), our data suggest that ATCC 33535 inhibition of OG1RF growth does not require cell-cell contact. This is in contrast to the contact-dependent mechanism of UA159 mutanobactin-mediated reduction in target cell viability. Furthermore, *E. faecalis* isolates do not recover by 24 h of co-culture with ATCC 33535, regardless of whether they produce gelatinase (Fig. S4B). This further supports our hypothesis that ATCC 33535 reduces target cell viability using a mechanism different from that of UA159.

### *S. mutans* UA159 and ATCC 33535 negatively impact *E. faecalis* biofilms

**Fig 6:**
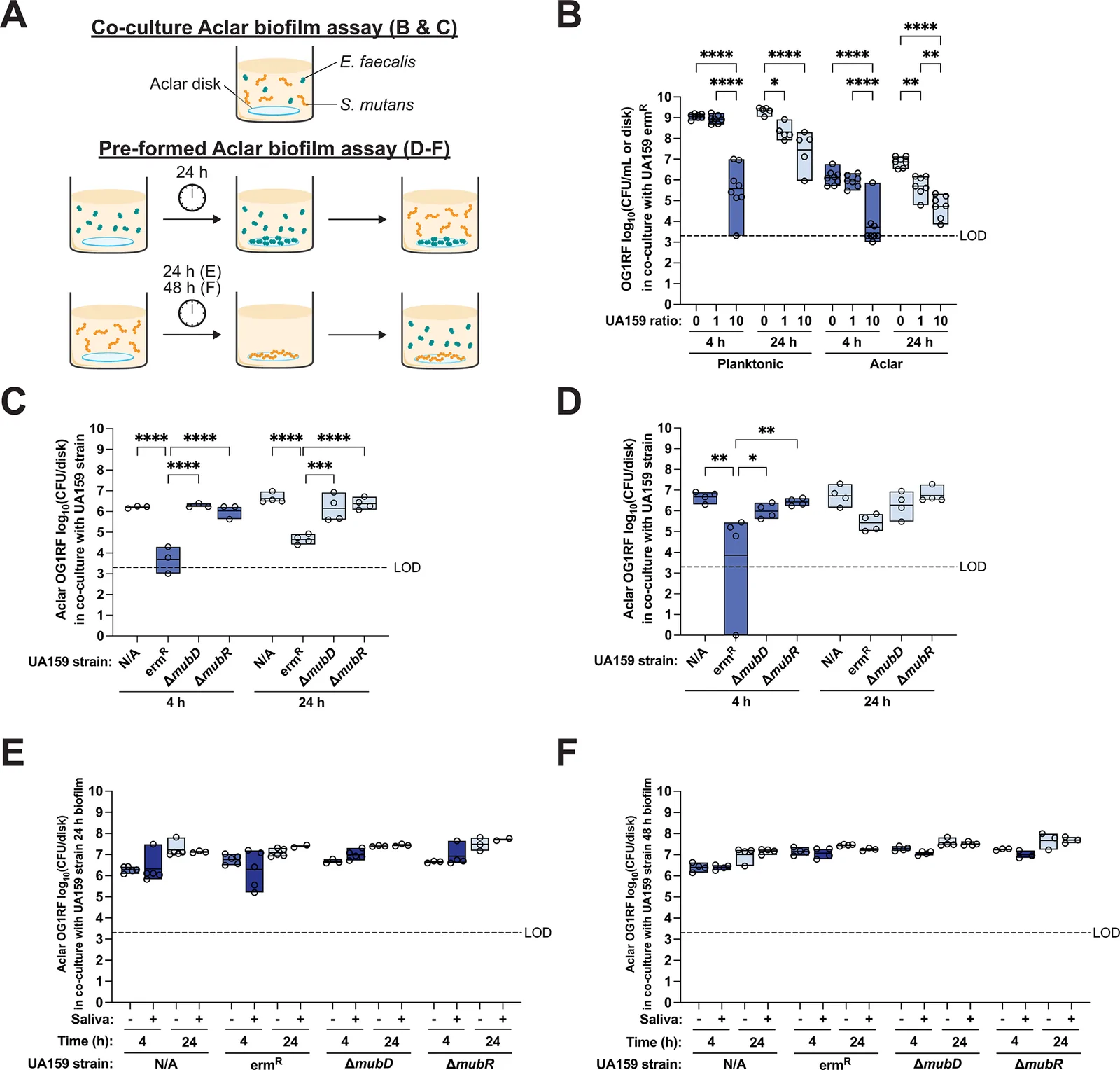
*S. mutans* UA159 prevents *E. faecalis* biofilm formation and reduces viability of biofilm-contained *E. faecalis*. (**A**) Experimental setups for the co-culture Aclar biofilm assays for panels **B** and **C** and the pre-formed Aclar biofilm assays for panels **D–F**. For the co-culture biofilm assays, Aclar disks are submerged in media inoculated with both *E. faecalis* and *S. mutans*. For the pre-formed biofilm assays, Aclar disks are cultured with either *E. faecalis* or *S. mutans* for 24–48 h before being transferred to an *E. faecalis* or *S. mutans* monoculture. (**B**) *E. faecalis* OG1RF CFU per milliliter for planktonic cells or CFU per Aclar disk for Aclar cells in co-culture with *S. mutans* UA159 erm^R^ at an indicated inoculum ratio of UA159 erm^R^ to one part OG1RF in BHI at 4 and 24 h. For each co-culture, *n* = 2 technical replicates for each of *n* = 7–8 biological replicates. (**C**) *E. faecalis* OG1RF CFU per Aclar disk in 1:10 co-culture with the indicated *S. mutans* UA159 strain in BHI at 4 and 24 h. For each co-culture, *n* = 2 technical replicates for each of *n* = 3–4 biological replicates. (**D**) *E. faecalis* OG1RF CFU per Aclar disk from the indicated *S. mutans* UA159 strain co-cultured with OG1RF biofilm pre-formed for 24 h on Aclar in BHI at 4 and 24 h. For each co-culture, *n* = 2 technical replicates for each of *n* = 4 biological replicates. Data point at *y* = 0 indicates no CFUs were detected. (**E**) *E. faecalis* OG1RF CFU per Aclar disk from OG1RF co-cultured with the indicated *S. mutans* UA159 strain biofilm pre-formed for 24 h on Aclar ± saliva coating in BHI + 1% sucrose at 4 and 24 h. For each co-culture, *n* = 2 technical replicates for each of *n* = 2–5 biological replicates. (**F**) *E. faecalis* OG1RF CFU per Aclar disk from OG1RF co-cultured with the indicated *S. mutans* UA159 strain biofilm pre-formed for 48 h on Aclar ± saliva coating in BHI + 1% sucrose at 4 and 24 h. For each co-culture, *n* = 2 technical replicates for each of *n* = 3–4 biological replicates. For panels **B–F**, two-way ANOVA was used for statistical analysis (*, *P* ≤ 0.05; **, *P* ≤ 0.01; ***, *P* ≤ 0.001; ****, *P* ≤ 0.0001; non-significant comparisons are not shown); for panels **E** and **F**, significantly higher mean values relative to *E. faecalis* monoculture comparisons are not shown. Each dot represents the mean of technical replicates; floating bars represent the minimum, mean, and maximum; dashed lines indicate the limit of detection (LOD).

*S. mutans* is typically found within dental plaque biofilms on the surface of teeth (62). Therefore, we sought to investigate interactions between *S. mutans* UA159 and *E. faecalis* OG1RF in the context of biofilms. We first tested whether OG1RF is able to form biofilms when co-cultured with UA159 using a well-established submerged substrate assay (79–81). For these assays, we co-cultured OG1RF and UA159 in the presence of an Aclar fluoropolymer disk and quantified CFU per milliliter in the planktonic phase as well as the number of biofilm CFUs per Aclar disk at 4 and 24 h (Fig. 6A). When inoculated in a 1:1 ratio with UA159, the number of OG1RF biofilm CFU/disk was reduced by three logs on average relative to monoculture OG1RF growth (Fig. 6B). When inoculated in a 1:10 ratio with UA159, the number of OG1RF biofilm CFU/disk was ~2 logs lower at both 4 and 24 h than when inoculated by itself (Fig. 6B). Planktonic OG1RF CFU per milliliter was also reduced when co-cultured with UA159 (Fig. 6B). We then tested whether UA159 mutanobactin genes are required to prevent OG1RF biofilm formation by performing 1:10 co-cultures of OG1RF with UA159 Δ*mubD* and Δ*mubR* in the presence of an Aclar disk. Neither the *mubD* nor *mubR* mutants reduced the viability of OG1RF biofilms or planktonic cells (Fig. 6C; Fig. S9A). Together, these results indicate that UA159 mutanobactin production significantly reduces OG1RF biofilm formation.

Next, we asked whether UA159 could reduce the viability of pre-formed *E. faecalis* biofilms. We grew OG1RF alone in the presence of an Aclar disk for 24 h, transferred the Aclar disk to a culture of UA159, and quantified the number of OG1RF biofilm CFU/disk at 4 and 24 h as well as CFU per milliliter of OG1RF in the planktonic phase (Fig. 6A). At 4 h after the transfer of the pre-formed OG1RF Aclar biofilm to a UA159 culture, we observed fewer OG1RF CFU/disk and planktonic CFU per milliliter than with OG1RF biofilms transferred to BHI, and the decrease in OG1RF biofilm and planktonic CFUs was dependent on *mubD* and *mubR* (Fig. 6D; Fig. S9B). Thus, UA159 mutanobactin production reduces the viability of biofilm-contained *E. faecalis* cells.

We then explored whether pre-formed UA159 biofilms prevent OG1RF biofilm formation on the same surface by growing UA159 in the presence of an Aclar disk for 24 h in media containing 1% sucrose, transferring the Aclar disk to a culture of OG1RF, and quantifying the number of OG1RF biofilm CFU/disk at 4 and 24 h as well as CFU per milliliter of OG1RF in the planktonic phase (Fig. 6A). While pre-formed UA159 biofilms slightly reduced the number of OG1RF CFU per milliliter in the planktonic phase by 24 h following UA159 Aclar biofilm transfer (Fig. S9C), OG1RF CFU/disk was not negatively impacted by the presence of UA159 on the disks, regardless of mutanobactin gene presence (Fig. 6E). We also tested OG1RF with UA159 biofilms pre-formed for 48 h and on Aclar disks coated in artificial saliva. However, none of these UA159 biofilms prevented OG1RF from forming biofilms on the same Aclar disk (Fig. 6E and F) or reduced planktonic OG1RF viability (Fig. S9C and D). In sum, these data show that *S. mutans* UA159 is effective in preventing *E. faecalis* biofilm formation and in reducing the viability of biofilm-contained *E. faecalis*, but pre-formed UA159 biofilms do not exclude *E. faecalis* from biofilms on the same surface. Furthermore, UA159 mutanobactin genes are required for its antibiofilm effects, supporting a role for mutanobactin in antagonizing *E. faecalis* biofilms.

**Fig 7:**
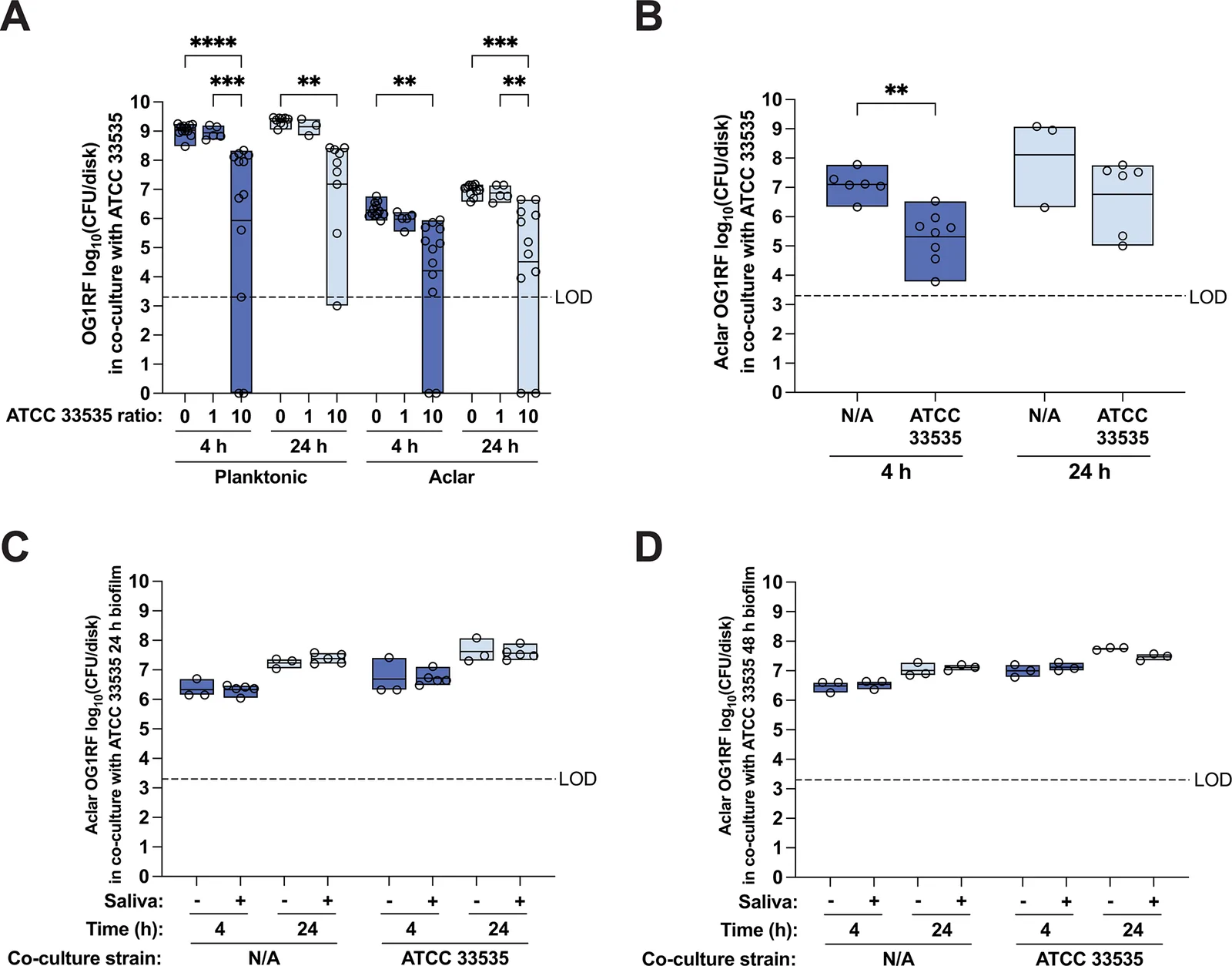
*S. mutans* ATCC 33535 prevents *E. faecalis* biofilm formation and reduces viability of biofilm-contained *E. faecalis*. (**A**) *E. faecalis* OG1RF CFU per milliliter for planktonic cells or CFU per Aclar disk for Aclar cells in co-culture with *S. mutans* ATCC 33535 at an indicated inoculum ratio of ATCC 33535 to 1 part OG1RF in BHI at 4 and 24 h. For each co-culture, *n* = 2 technical replicates for each of *n* = 3–12 biological replicates. (**B**) *E. faecalis* OG1RF CFU per Aclar disk from *S. mutans* ATCC 33535 in co-culture with OG1RF biofilm pre-formed for 24 h on Aclar in BHI at 4 and 24 h. For each co-culture, *n* = 2 technical replicates for each of *n* = 3–8 biological replicates. (**C**) *E. faecalis* OG1RF CFU per Aclar disk from OG1RF co-cultured with *S. mutans* ATCC 33535 24 h biofilm pre-formed on Aclar ± saliva coating in BHI + 1% sucrose at 4 and 24 h. For each co-culture, *n* = 2 technical replicates for each of *n* = 3–5 biological replicates. (**D**) *E. faecalis* OG1RF CFU per Aclar disk from OG1RF co-cultured with *S. mutans* ATCC 33535 48 h biofilm pre-formed on Aclar ± saliva coating in BHI + 1% sucrose at 4 and 24 h. For each co-culture, *n* = 2 technical replicates for each of *n* = 3 biological replicates. For panels **A–D**, two-way ANOVA was used for statistical analysis (**, *P* ≤ 0.01; ***, *P* ≤ 0.001; ****, *P* ≤ 0.0001); non-significant comparisons are not shown. Each dot represents the mean of technical replicates; floating bars represent the minimum, mean, and maximum; dashed lines indicate the limit of detection (LOD).

Finally, we assessed whether the non-mutanobactin-producing *S. mutans* strain ATCC 33535 impacts *E. faecalis* biofilms. When ATCC 33535 and *E. faecalis* OG1RF were co-cultured in the presence of an Aclar disk, OG1RF formed fewer CFU/disk and had fewer planktonic CFU per milliliter at 4 and 24 h when ATCC 33535 was co-inoculated in a 10:1 ratio with OG1RF compared to an OG1RF monoculture (Fig. 7A). The number of viable cells in pre-formed OG1RF biofilms was also reduced when cultured with ATCC 33535 compared to OG1RF biofilms cultured in BHI (Fig. 7B), and viable planktonic OG1RF in the same assay was reduced at 4 h (Fig. S9E). Pre-formed ATCC 33535 biofilms, for 24 and 48 h with and without Aclar coated in artificial saliva, did not reduce the number of viable OG1RF planktonic cells (Fig. S9F and G) nor did they decrease OG1RF cells on the same Aclar disk (Fig. 7C and D) compared to OG1RF grown in BHI. Similar to our UA159 findings, these results show that planktonic ATCC 33535 prevents *E. faecalis* biofilm formation and reduces biofilm-contained *E. faecalis*, but pre-formed ATCC 33535 biofilms do not exclude *E. faecalis* from forming biofilms on the same surface. The biofilm-dependent differences in competitive ability for both strains of *S. mutans* are suggestive of underlying mechanisms that regulate the expression of factors that mediate competition under specific conditions, such as biofilm growth.

## DISCUSSION

*E. faecalis* is a well-characterized pathobiont that is understudied in the context of the oral cavity despite its prevalence in root canal infections (24–33). Our study aimed to explore microbial factors that could influence *E. faecalis* competition and persistence within the oral cavity. Our experiments revealed that multiple strains of the oral pathogen *S. mutans* reduce the viability of *E. faecalis* OG1RF (Fig. 3A) as well as multiple other gram-positive species, including ESKAPE pathogens *E. faecium* and *S. aureus* (Fig. 1B and 3C) and antibiotic-resistant strains of *E. faecalis* (Fig. 3B; Fig. S2 and S3E). We also determined that UA159 reduction of target cell viability is dependent on genes responsible for producing mutanobactin (Fig. 4C and D), a cyclic lipopeptide that promotes *S. mutans* oxygen and hydrogen peroxide stress tolerance (57–59), inhibits the *C. albicans* yeast-to-mycelium transition (56, 60), and modulates the immune response of human cells (58). While synthesized mutanobactin was previously shown to have inhibitory activity against several oral bacteria in a 64 h assay (61), our work provides strong genetic evidence that *in vivo* mutanobactin production by *S. mutans* mediates competition with other bacterial species on a much shorter time scale (4–6 h) by increasing target cell permeability to reduce target cell viability. Our work also expands the known target range of mutanobactin to include clinically relevant gram-positive ESKAPE pathogens. Overall, this work describes interbacterial functions for mutanobactin production by *S. mutans*, revealing a new interaction between clinically relevant bacteria that occupy the oral cavity.

In addition to UA159, *S. mutans* strains UA140 and ATCC 25175 both encode genes for the production of mutanobactin (57). However, the timing of growth, and thus mutanobactin expression, differs between UA159 and UA140. In an experiment by Wu et al., UA159 exponential phase began at 3 h and ended at 7 h while UA140 exponential phase began later at 6 h and ended at 10 h, with *mub* expression for each strain corresponding accordingly (57). The later timing of UA140 *mub* expression may explain why we did not observe significant reduction in *E. faecalis* OG1RF viability by UA140 (Fig. 3A), as OG1RF *gelE* expression is induced during later stages of growth (53), prior to induction of *mub* expression. Therefore, differences in *mub* expression levels or other factors may account for the differences in reduction of target cell viability observed between ATCC 25175 and UA159, as well.

ATCC 33535 does not produce mutanobactin yet reduces target cell viability longer and at a larger magnitude than any of the mutanobactin-producing strains of *S. mutans* (Fig. 3A). While the factors that contribute to ATCC 33535 reduction of target cell viability remain unknown, our work suggests that multiple mechanisms could be involved. Spent media from ATCC 33535 inhibited *E. faecalis* growth (Fig. S8B) but not to the extent that ATCC 33535 in co-culture reduced *E. faecalis* viability (Fig. 3A). This suggests that ATCC 33535 may use both contact-dependent and contact-independent mechanisms to reduce target cell viability. An alternative model is that any inhibitory compound produced by ATCC 33535 may be disrupted by the process of generating spent media. However, analysis of the ATCC 33535 genome using antiSMASH revealed multiple regions predicted to encode secondary metabolite clusters (Table 2), further supporting the idea that multiple inhibitory mechanisms may be at play. Our ATCC 33535 results highlight the diversity of streptococcal natural products that can mediate interbacterial competition, with contact-dependent and contact-independent mechanisms potentially contributing to differential inhibition of specific microbial targets in specific oral niches.

Without production of the extracellular protease GelE, *E. faecalis* does not fully recover from mutanobactin-mediated killing by 24 h of co-culture with UA159 (Fig. 2), but GelE-negative strains do partially recover to near or above the limit of detection (Fig. 2A). This is consistent with the timing of mutanobactin gene expression in UA159, where expression is at its maximum at the end of the exponential phase at 6 h of growth, followed by a dramatic decrease in expression once cells enter the stationary phase (57). Therefore, *E. faecalis* overcoming a reduction in viability mediated by UA159 is likely influenced by the temporal expression of both enterococcal GelE and UA159 mutanobactin. Since expression of *gelE* is controlled by the *fsr* quorum sensing system (53), *gelE* expression should be minimal at early time points (0–6 h) in our assays since *E. faecalis* cell density is low when co-cultured with UA159. One possible explanation for the requirement of GelE for full recovery is that GelE facilitates the outgrowth of surviving *E. faecalis* cells only after mutanobactin expression decreases after UA159 reaches the stationary phase. This hypothesis is supported by our data demonstrating that *fsr*-independent induction of *gelE* expression at the beginning of co-culture did not prevent killing of OG1RF at 4 and 6 h of co-culture with UA159 (Fig. 2B). The stability of mutanobactin in *S. mutans* cultures and how that may contribute to *E. faecalis* recovery are unclear. Although mutanobactin has been extracted from *S. mutans* cultured for 48 h in a bioreactor (56), expression of *mub* genes in a continuous growth system may not match our culture system. Regardless, other factors, streptococcal and/or enterococcal, may also contribute to *E. faecalis* recovery.

We observed that other species recover from reduction in viability by 24 h of co-culture with UA159 (Fig. 1B; Fig. S3D and F) and that four extracellular proteases of *S. aureus* do not contribute to this recovery (Fig. S3E). Other *S. aureus* factors may contribute to recovery from mutanobactin-mediated killing, but it is likely that recovery is at least in part due to decreased mutanobactin production by UA159. Enterococcal GelE may act directly on mutanobactin to degrade or inhibit it or could process a specific component of the *E. faecalis* cell surface (such as a receptor or membrane protein) that is required for inhibition by mutanobactin. The latter could explain why the *S. aureus* extracellular proteases do not impact recovery from reduction in viability: if an *S. aureus* cell surface factor is required for mutanobactin-mediated reduction in viability, then perhaps the *S. aureus* proteases do not process this factor in the same manner as GelE. It is also possible that mutanobactin induces transcriptional changes in *E. faecalis* that promote tolerance over time. Future work will define the mechanism by which GelE contributes to recovery from mutanobactin-mediated killing.

While *E. faecalis* is frequently isolated from the oral cavity and is found in saliva, it is not commonly found within dental plaques where *S. mutans* is observed (82, 83). This is despite the ability of enterococci to form biofilms on uncolonized and pre-colonized enamel slabs and integrate into *in vitro* six-species oral biofilms that contain *S. mutans* (84, 85). Toxins that are released extracellularly, such as the one(s) produced by *S. mutans* ATCC 33535, may diffuse to other niches in the oral cavity, thereby impacting viability and biofilm formation by other bacteria such as *E. faecalis*. Conversely, inhibitory strategies that require cell-cell contact, as we have shown with mutanobactin, may affect *E. faecalis* viability through broader alterations in polymicrobial community structure. The biological significance of mutanobactin within the oral cavity may also be relevant for other bacteria that reside in dental plaque or other oral cavity sites. Future work will determine the spatial and temporal dynamics of *S. mutans* compound production with regard to antibacterial activity toward *E. faecalis* and other oral bacteria.

We were surprised that *S. mutans* UA159 biofilms did not exclude *E. faecalis* from forming dual-species biofilms on Aclar (Fig. 6E and F) and that inhibition of planktonic *E. faecalis* by UA159 biofilms was only slight (Fig. S9C and D), even when UA159 biofilms were cultured with 1% sucrose or artificial saliva (Fig. 4C; Fig. 6E and F). One explanation for this observation is that mutanobactin expression by biofilm-contained UA159 may be reduced compared to planktonic UA159. In support of this idea, studies measuring mutanobactin gene expression have shown that mutanobactin genes are regulated differently, depending on the context (72, 86). ATCC 33535 biofilms also failed to exclude *E. faecalis* from forming biofilms on the same surface (Fig. 7C and D), suggesting that the factors that mediate ATCC 33535 competition with *E. faecalis* may also be downregulated in biofilms. The ability of *E. faecalis* to form biofilms despite the presence of an *S. mutans* biofilm could explain why *E. faecalis* is able to occupy the oral cavity in the presence of *S. mutans* since *S. mutans* is primarily found in biofilms rather than as planktonic cells. The mechanism underlying biofilm formation by *E. faecalis* and *S. mutans* on the same surface is unclear, but we hypothesize that *S. mutans* and *E. faecalis* biofilms are spatially separated from each other, thereby reducing cell-cell contact between the two species. It is also possible that *S. mutans* does not produce mutanobactin within biofilms, which would be consistent with UA159 production of mutanobactin coinciding with exponential growth (57). Additional studies are needed to delineate the spatiotemporal dynamics of *S. mutans* and *E. faecalis* biofilm formation in the oral cavity and how *E. faecalis* is able to establish itself as an endodontic pathogen.

**Fig 8:**
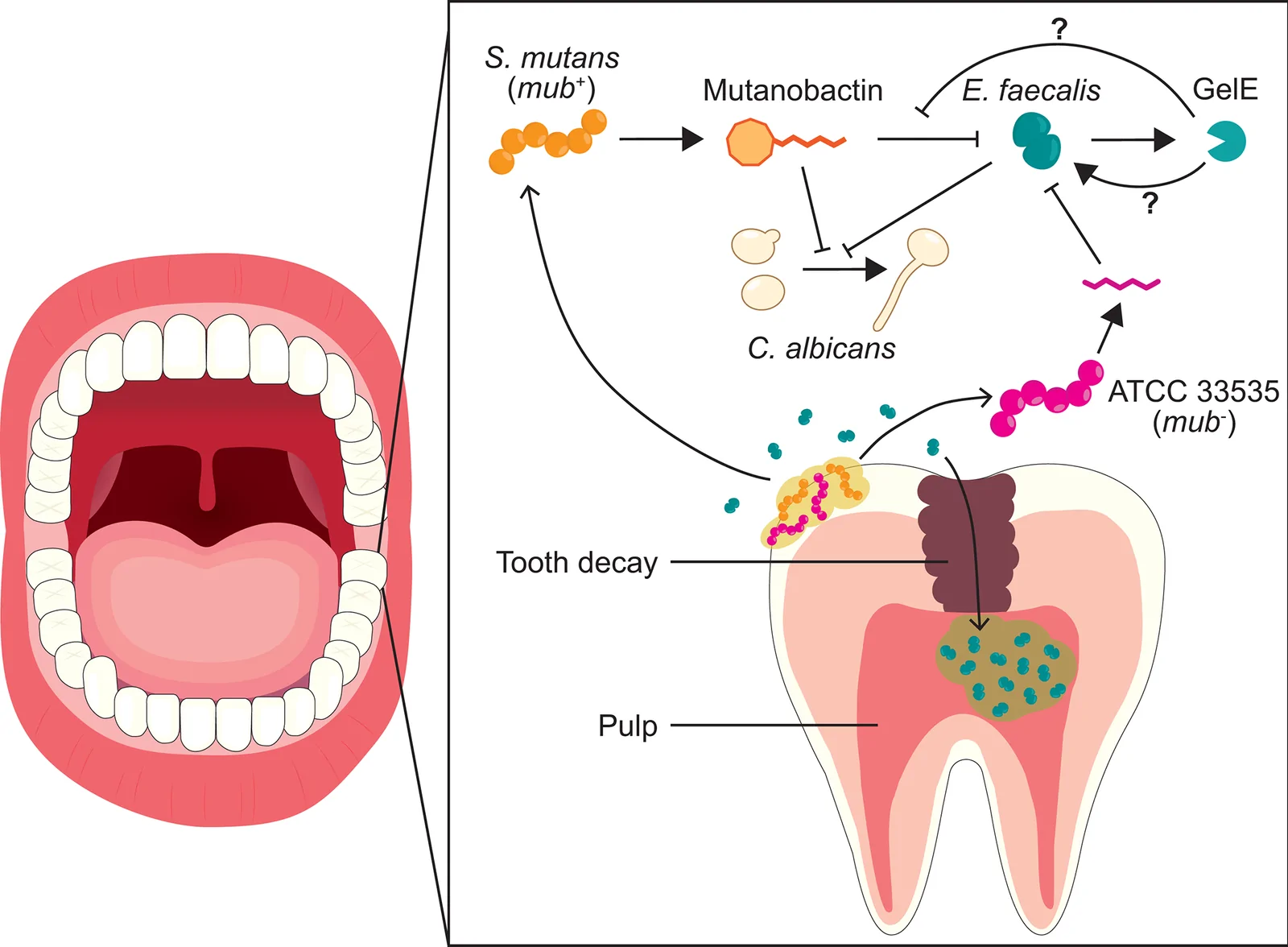
Proposed model of *S. mutans* and *E. faecalis* interactions within the oral cavity. *S. mutans* and *E. faecalis* are both found in the oral cavity and have both been detected in infected root canals. *S. mutans mub*^+^ strains produce mutanobactin, which kills *E. faecalis* in a contact-dependent manner. *E. faecalis* secretion of GelE promotes recovery from killing or prevents mutanobactin from killing. *S. mutans mub*^−^ strain ATCC 33535 does not produce mutanobactin but releases an uncharacterized compound that kills *E. faecalis* in a contact-independent manner. *C. albicans* hyphal morphogenesis is inhibited by *E. faecalis* (87–89) and by mutanobactin (56, 60). Biofilm-contained *S. mutans* does not inhibit *E. faecalis*, which may allow *E. faecalis* to persist in the presence of *S. mutans* in the oral cavity.

We have summarized our results in the context of previous work in the model presented in Fig. 8. We propose the following model: *S. mutans* production of mutanobactin kills *E. faecalis* while *E. faecalis* GelE secretion promotes recovery from killing, and both mutanobactin-producing and non-mutanobactin-producing strains of *S. mutans* in dental plaque biofilms do not kill *E. faecalis*, thereby reducing competition with *E. faecalis* and leading to enterococcal colonization of root canals. This expands upon our current knowledge of mutanobactin-mediated microbial antagonism and *E. faecalis* interspecies interactions as both are known to inhibit *C. albicans* hyphal morphogenesis (56, 60, 87–89). Future studies testing this model will elucidate the spatiotemporal dynamics of *S. mutans* interactions with *E. faecalis* in the oral cavity.

This work describes a novel interaction between two bacteria found in the oral cavity, *S. mutans* and *E. faecalis*. Our findings also revealed antibacterial effects of streptococcal natural products on various bacterial species, including ESKAPE pathogens. We identified a mutanobactin- and contact-dependent strategy that *S. mutans* strains use to reduce target cell viability and uncovered another strategy that likely involves a different mechanism of killing target cells that is not completely contact dependent. The natural product-mediated antibiofilm effects we observed by *S. mutans* against *E. faecalis* serve as a starting point to develop new treatments to treat this clinically important pathobiont that causes widespread infections in humans. While mutanobactin has previously been characterized, this work is the first demonstration of its widespread antibacterial activity against antibiotic-resistant pathogens, highlighting the functional diversity of metabolites produced by microbes. The total synthesis of streptococcal mutanobactin has been described (61, 90), providing a framework for the potential development of mutanobactin as a treatment for infections, including those resistant to antibiotics. New and unknown streptococcal natural products may also be exploited for therapeutic control of bacterial infections. In this age of increased need for antibiotic discovery, our work underscores the benefits of identifying novel natural products from well-characterized species and investigating known natural products for potential antimicrobial activity.

## MATERIALS AND METHODS

### Bacterial strains, plasmids, media, and growth conditions

Strains used in this study are listed in Table 1. Plasmids used in this study are listed in Table 2. Strains were grown in BHI medium (per liter: 37 g Difco BHI [BD]) unless otherwise noted. OG1RF strains were grown in tryptic soy broth without added dextrose (TSB-D) medium (per liter: 27.5 g Bacto tryptic soy broth without dextrose [BD]) where noted. *E. coli* strains used for cloning were grown in LB medium (per liter: 25 g LB broth [Electron Microscopy Sciences]; or 10 g tryptone [Fisher Scientific], 5 g yeast extract [Bacto Difco or Electron Microscopy Sciences], 1 g glucose, 5 g NaCl) or BHI medium. When needed, antibiotics were added to media at the following concentrations: fusidic acid, 25 μg/mL for OG1RF; erythromycin, 10 μg/mL for OG1RF, 5 μg/mL for UA159; tetracycline, 10 μg/mL for OG1RF and *E. coli*; and spectinomycin, 150 μg/mL for *E. coli*, 250 μg/mL for UA159. When needed, the peptide pheromone cCF10 (50 ng/mL, Mimotopes) was added to the media. When needed, 1% (wt/vol) sucrose was added to the media. Growth medium was solidified using 1% agar when needed. For gelatin plates, 3% (wt/vol) gelatin was added to BHI agar. *Enterococcus* spp. and *Streptococcus* spp. were grown at 37°C in 5% CO_2_ without agitation unless otherwise noted. *E. coli*, *Staphylococcus* spp., *K. pneumoniae*, *A. baumannii*, *P. aeruginosa*, and *E. aerogenes* were grown at 37°C with shaking unless otherwise noted.

### DNA synthesis and sequencing

Primers used in this study are listed in Table S3 and were synthesized by Thermo Fisher Scientific or Integrated DNA Technologies (Coralville, IA, USA). The full pGelE plasmid was confirmed by whole plasmid sequencing by Plasmidsaurus using Oxford Nanopore Technology with custom analysis and annotation. *S. mutans* UA159 erm^R^, Δ*mubD*, and Δ*mubR* strains were confirmed by whole genome sequencing performed by SeqCoast Genomics (Portsmouth, NH, USA) using Illumina short-read sequencing with 150 bp paired-end reads. PCR to amplify constructs for cloning and strain construction were performed using PfuUltra II or PfuUltra II Fusion HS DNA Polymerase (Agilent Technologies) or Q5 High-Fidelity DNA Polymerase (NEB). Diagnostic PCR was performed using Taq or OneTaq DNA Polymerase (NEB).

### Construction of *S. mutans* UA159 strains

Mutagenesis was performed according to an established protocol (91) to insert an erythromycin resistance cassette in the intergenic region between UA159 *ldh* (SMU_1115) and SMU_1116c to create the UA159 erm^R^ strain or to replace UA159 *mubD* or *mubR*. For the *ldh*/SMU_1116c IG::*erm* strain, ~600 to 800 bp of the upstream and downstream regions flanking the *ldh*/SMU_1116c intergenic insertion site was amplified by PCR using primer pairs JW288/JW289 and JW292/JW293, respectively. For the Δ*mubD::erm* strain, ~900 to 1,000 bp of the upstream and downstream regions flanking *mubD* was amplified using primer pairs JW365/JW366 and JW369/JW370, respectively. For the Δ*mubR::erm* strain, ~1,000 bp of the upstream and downstream regions flanking *mubR* was amplified using primer pairs JW371/JW372 and JW375/JW376, respectively. For all mutants, the erm^R^ cassette from pMSP3535 was amplified using primer pair JW290/JW291. For *ldh*/SMU_1116c IG::*erm* and Δ*mubD::erm*, the resulting PCR products were treated with DpnI and cleaned over a silica column (Epoch). The upstream, downstream, and erm^R^ PCR products for each strain were then spliced together by overlap extension PCR using the forward primer used to amplify the upstream region and the reverse primer used to amplify the downstream region. The resulting linear mutagenic DNA fragments were transformed into UA159, and transformants were selected using erythromycin. Strains were confirmed by PCR using primers flanking the deleted gene, followed by whole genome sequencing.

### Construction of the pJW1016 *gelE* expression plasmid

*E. faecalis* OG1RF *gelE* was PCR amplified from genomic DNA using primer pair JW386/RYI011, which added flanking BamHI and NheI restriction sites. The amplified *gelE* and pJW76 were digested with BamHI and NheI (NEB), then digested *gelE* was ligated into digested pJW76 using T4 DNA ligase (NEB). The ligation reaction was transformed into chemically competent DH5α. Τransformants were selected using tetracycline and confirmed by PCR using primer pair JW50/JW199 followed by whole plasmid sequencing. Electrocompetent OG1RF Δ*gelE* was transformed with pJW1016; transformants were selected using tetracycline; and the strain was confirmed by PCR.

### Construction of pJW1204 and pJW1205 *mub* transcriptional reporter plasmids

*S. mutans* UA159 *mubR* and *mubX* promoter regions were amplified using primer RYI022/RYI023 and RYI024/RYI025 primer pairs, respectively. pTCV-GFP-Spec was amplified using primer pair RYI018/RYI019. The pTCV-GFP PCR product was treated with DpnI (NEB). The *mubR* and *mubX* promoters were assembled with pTCV-GFP-Spec by Gibson assembly using NEBuilder HiFi Assembly Master Mix (NEB). The Gibson reactions were transformed into chemically competent DH5α. Transformant candidates were selected using spectinomycin and confirmed by PCR using primer pair RYI020/RYI021 followed by whole plasmid sequencing. pJW1204 and pJW1205 were transformed into *S. mutans* UA159; transformants were selected using spectinomycin; and strains were confirmed by PCR.

### Co-culture assay

Overnight cultures of target strains were diluted to 10^7^ CFU/mL or OD_600_ of 0.01 in BHI in a U-bottom 96-well plate. Overnight cultures of competing strains were inoculated with the target strain at the corresponding CFU per milliliter or OD_600_ for 1:1, 1:2, 1:5, or 1:10 co-cultures. Co-cultures were incubated at 37°C in 5% CO_2_. For each time point, samples of co-cultures were removed, diluted in potassium-based phosphate-buffered saline (KPBS; 10 mM, pH 7.4), and spotted on BHI agar to quantify CFU per milliliter of each strain in co-culture. For CFU enumeration, fusidic acid and/or incubation without 5% CO_2_ was used to select for OG1RF strains; incubation without 5% CO_2_ was used to select for all other target strains; and erythromycin was used to select for UA159 erm^R^, Δ*mubD*, and Δ*mubR* strains.

### Measurement of culture pH

Monocultures were set up by diluting overnight to 10^7^ CFU/mL for *E. faecalis* OG1RF or 10^8^ CFU/mL for *S. mutans* UA159 in BHI in a glass culture tube. Co-cultures of OG1RF and UA159 were set up as in a 1:10 ratio in BHI in glass culture tubes. Cultures were incubated at 37°C in 5% CO_2_ for 24 h. The pH of cultures was measured using ColorpHast indicator strips (pH 4.0–7.0) (EM Science).

### Culture viability at different pH

Overnight cultures of *E. faecalis* OG1RF were diluted to 10^7^ CFU/mL in BHI adjusted to pH 2, 4, 5, 6, and 7. BHI pH was adjusted by adding 5 M HCl and 5 M NaOH to 2× BHI, then adjusting the volume to 1× BHI with water, followed by filter sterilization using a 0.22 μm filter. Cultures were incubated at 37°C. For each time point, samples of cultures were removed, diluted in KPBS (10 mM, pH 7.4), and spotted on BHI agar to quantify CFU per milliliter of each strain in co-culture.

### Gelatinase assay

Overnight cultures of *E. faecalis* clinical isolates were spotted onto BHI agar supplemented with 3% (wt/vol) gelatin and incubated overnight (~24 h) at 37°C. Plates were imaged using a ProteinSimple Cell Biosciences FluorChem FC3 System imager. The radius of the opaque gelatinase halo for each strain was measured from the edge of the colony to the edge of the gelatinase halo using FIJI (92).

### *mub* transcriptional reporter analysis

Co-cultures of *E. faecalis* OG1RF and *S. mutans* UA159 carrying the pJW104 *mubR* reporter plasmid, the pJW1205 *mubX* reporter plasmid, or the pGFP-Spec promoterless vector control were set up as described in the co-culture assay methods above in glass culture tubes. Monocultures of the *S. mutans* UA159 strains were included as controls. Cultures were incubated at 37°C in 5% CO_2_. For each time point, samples of cultures were removed, pelleted at 8,000 × *g* for 2 min, resuspended in KPBS (10 mM, pH 7.4), and aliquoted into a black-wall 96-well plate. The absorbance at 600 nm (*A*_600_) and GFP (480 nm excitation/510 nm emission) for each culture were measured using a BioTek Synergy H1 plate reader. To calculate *mub* reporter activity, GFP values (reporting promoter activity) were normalized to *A*_600_ values.

### CPRG cell membrane permeability assay

CPRG assays were performed according to previously described methods (76, 77). Overnight cultures of OG1RF pCJK205 grown in TSB-D-Erm^10^ were diluted to 10^7^ CFU/mL in TSB-D with CPRG (25 μg/mL) in a glass culture tube and incubated at 37°C with 5% CO_2_ for 24 h. For antibiotic CPRG assays, subinhibitory concentrations of daptomycin (8 μg/mL + 50 µg/mL CaCl_2_) and ciprofloxacin (0.5 μg/mL) were added to the media. For co-culture CPRG assays, overnight cultures of UA159 strains grown in TSB-D-Erm^10^ were inoculated with OG1RF pCJK205 at 10^8^ CFU/mL. CPRG cleavage was measured by pelleting cells at 8,000 × *g* for 2 min, transferring the supernatant to a flat-bottom 96-well plate, and measuring *A*_570_ using a BioTek Synergy H1 plate reader.

### Supernatant growth assay

Overnight cultures of strains were diluted to 10^8^ CFU/mL in BHI and incubated for 4 h at 37°C with 5% CO_2_. For co-culture supernatants, OG1RF was inoculated at 10^7^ CFU/mL with UA159 or ATCC 33535 at 10^8^ CFU/mL. Cell-free supernatants were obtained by removing cells via centrifugation at 8,000 × *g* for 2 min followed by filter sterilization of supernatants using a 0.45 μm filter. Overnight cultures of OG1RF were diluted to 10^7^ CFU/mL in a 50/50 mixture of BHI and cell-free supernatant and incubated for 4 h at 37°C with 5% CO_2_. To quantify OG1RF CFU per milliliter, cultures were diluted in KPBS (10 mM, pH 7.4) and spotted on BHI agar.

### Aclar biofilm assays

Overnight cultures of OG1RF or UA159 strains were diluted to 10^7^ or 10^8^ CFU/mL, respectively, in BHI (+1% sucrose for UA159) in a 48-well plate containing a sterile 5 mm diameter Aclar fluoropolymer disk (Electron Microscopy Sciences). For co-culture biofilm assays, overnight cultures of UA159 strains were inoculated with OG1RF at 10^7^ or 10^8^ CFU/mL and incubated at 37°C with 5% CO_2_. For pre-formed biofilm assays, OG1RF and UA159 biofilms were incubated at 37°C with 5% CO_2_ overnight, then Aclar disks were washed 3× in KPBS (10 mM, pH 7.4) before transferring to fresh BHI containing 10^7^ OG1RF or 10^8^ CFU/mL UA159 strains and incubated at 37°C with 5% CO_2_. At 4 and 24 h, planktonic cells were removed, diluted in KPBS, and spotted on BHI agar to quantify CFU per milliliter of each strain. To quantify CFUs of Aclar biofilm cells at 4 and 24 h, Aclar disks were removed, washed 3× in KPBS, transferred to a 1.5 mL microcentrifuge tube containing 1 mL KPBS, and vortexed for 5 min at 2,500 rpm using a multi-tube vortexer, and then cells were diluted in KPBS and spotted on BHI agar. For CFU enumeration, fusidic acid and incubation without 5% CO_2_ were used to select for OG1RF strains, and erythromycin was used to select for UA159 strains.

### Whole genome sequencing and genome analysis

Whole genome sequencing of *S. mutans* strains UA159B, Ingbritt, and ATCC 33535 was performed by SeqCoast Genomics using Illumina short-read sequencing with 150 bp paired-end reads. The UA159B genome sequence was compared to the available UA159 genome sequence (NC_004350.2) using breseq (93). Acquired and available *S. mutans* genomes were queried for *mubR* and *mubD* homologs using NCBI BLAST. The *mubR* homolog in ATCC 25175 (taxonomy ID 1257041) is locus tag D820_RS03580/D820_06113 on contig008 of reference sequence NZ_AOCB01000008.1. The *mubR* and *mubD* genes in UA159 and UA140 were identified from published work (57). The ATCC 33535 genome was analyzed for secondary metabolite regions by antiSMASH v7.1.0 using strictness “relaxed” (65).

### Data analysis

CFU per milliliter data were log_10_ transformed before performing statistical analysis. Limit of detection (LOD) for CFU per milliliter was determined by calculating CFU per milliliter based on the smallest number of colonies (1) that could be detected at the lowest dilution (10^−1^) plated. GraphPad Prism was used to generate graphs and conduct statistical analyses. Graphs and figures were assembled and refined in Adobe Illustrator.

## Data Availability

Genome sequencing files for *S. mutans* strains UA159, UA159B, Ingbritt, and ATCC 33535 have been deposited with NCBI Genome under BioProject ID PRJNA974474.

## SUPPLEMENTAL MATERIAL

Supplemental Material mbio.01623-26-s0001.pdf Figures S1-S9; Tables S1-S3.

ASM does not own the copyrights to Supplemental Material that may be linked to, or accessed through, an article. The authors have granted ASM a non-exclusive, world-wide license to publish the Supplemental Material files. Please contact the corresponding author directly for reuse.
