# Supplementary material for: Streptococcal mutanobactin mediates interspecies competition with gram-positive bacteria
Source: mBio. 2026 Oct 6;17(11):e01623-26. doi: 10.1128/mbio.01623-26 (PMC13753741; doi:10.1128/mbio.01623-26)
Supplement: Supplementary file 1 [file mbio.01623-26-s0001.pdf]

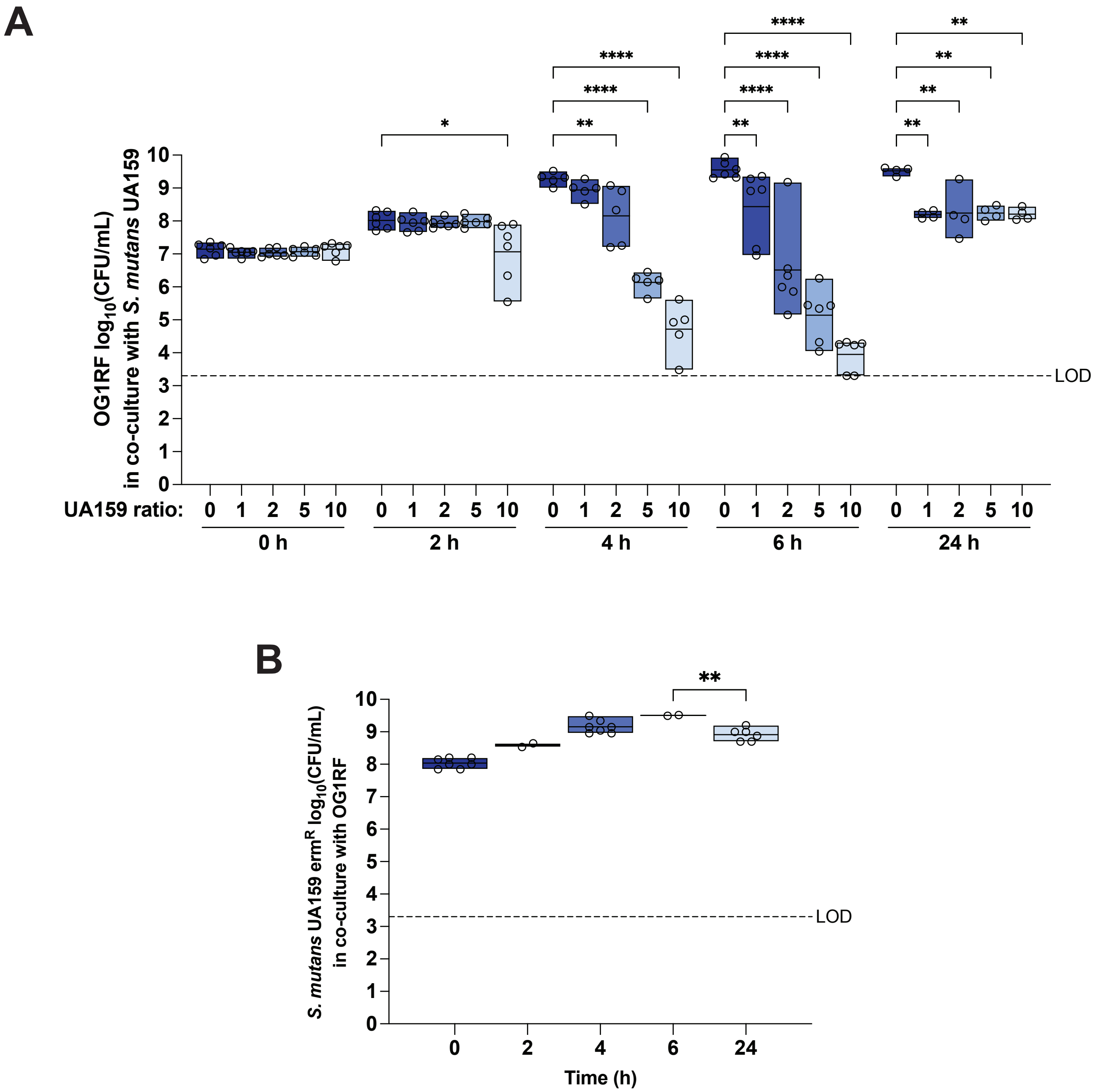

Fig. S1. *S. mutans* UA159 reduces viability of *E. faecalis* in a dose-dependent manner. A. *E. faecalis* OG1RF CFU/mL in co-culture with indicated *S. mutans* UA159 erm^R at indicated inoculum ratio of UA159 erm^R to 1 part OG1RF in BHI at 0, 2, 4, 6, and 24 h. For each culture, n = 2 technical replicates for each of n = 4-6 biological replicates. Two-way ANOVA was used for statistical analysis (*, p ≤ 0.05; **, p ≤ 0.01; ****, p ≤ 0.0001; not significant and significantly higher mean relative to 0 h comparisons are not shown). **B.** *S. mutans* UA159 erm^R CFU/mL in 10:1 co-culture with *E. faecalis* OG1RF in BHI at 0, 2, 4, 6, and 24 h. For each culture, n = 2 technical replicates for each of n = 2-7 biological replicates. One-way ANOVA was used for statistical analysis (**, p ≤ 0.01; not significant and significantly higher mean relative to 0 h comparisons are not shown). For panels A and B, each dot represents the mean of technical replicates; floating bars represent the minimum, mean, and maximum; dashed lines indicate limit of detection (LOD).

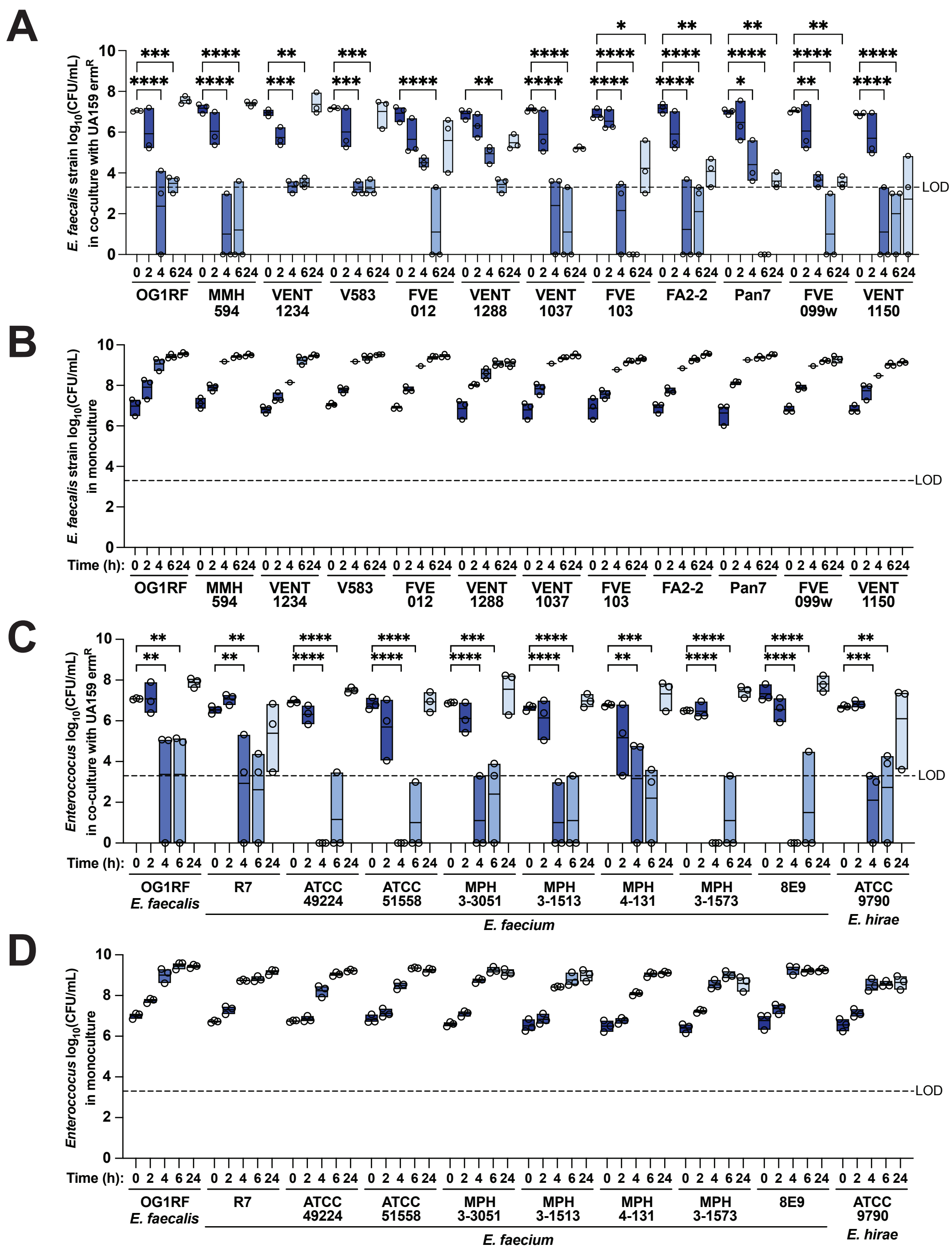

Fig. S2. *S. mutans* UA159 reduces viability of *E. faecalis* clinical isolates and *Enterococcus* spp. strains. A. CFU/mL of indicated *E. faecalis* clinical isolates in 1:10 co-culture with *S. mutans* UA159 erm^R in BHI at 0, 2, 4, 6, and 24 h. For each culture, n = 2 technical replicates for each of n = 1-3 biological replicates. **B.** CFU/mL of indicated *E. faecalis* clinical isolates in BHI monoculture at 0, 2, 4, 6, and 24 h performed in parallel with the co-culture experiment shown in panel A. For each culture, n = 2 technical replicates for each of n = 1-3 biological replicates. **C.** CFU/mL of indicated *Enterococcus* spp. strains in 1:10 co-culture with *S. mutans* UA159 erm^R in BHI at 0, 2, 4, 6, and 24 h. For each culture, n = 2 technical replicates for each of n = 3 biological replicates. **D.** CFU/mL of indicated *Enterococcus* spp. strains in BHI monoculture at 0, 2, 4, 6, and 24 h performed in parallel with the co-culture experiment shown in panel C. For each culture, n = 2 technical replicates for each of n = 3 biological replicates. For panels A-D, two-way ANOVA was used for statistical analysis (*, p ≤ 0.05; **, p ≤ 0.01; ***, p ≤ 0.001; ****, p ≤ 0.0001; not significant and significantly higher mean relative to 0 h comparisons are not shown); each dot represents the mean of technical replicates; floating bars represent the minimum, mean, and maximum; dashed lines indicate limit of detection (LOD); data points at y = 0 indicate no CFUs were detected.

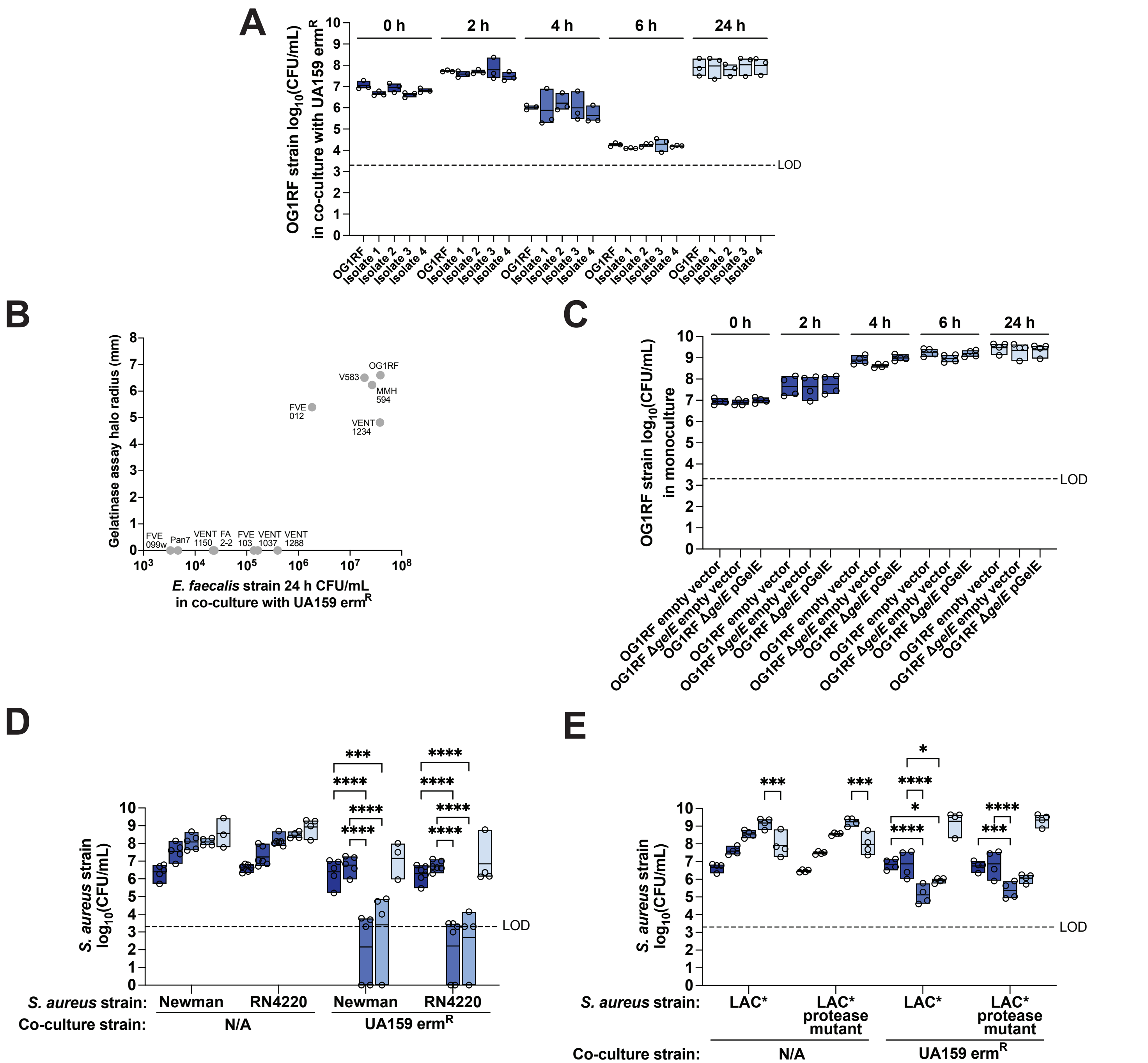

Fig. S3. ***E. faecalis*** **gelatinase activity is required for recovery from reduction of viability by** ***S. mutans*** **UA159.**
A. CFU/mL of *E. faecalis* OG1RF and OG1RF 24 h co-culture isolates in 1:10 co-culture with *S. mutans* UA159 erm^R in BHI at 0, 2, 4, 6, and 24 h. For each culture, n = 2 technical replicates for each of n = 3 biological replicates. **B.** CFU/mL of indicated *E. faecalis* OG1RF strain monoculture in BHI at 0, 2, 4, 6, and 24 h. For each culture, n = 2 technical replicates for each of n = 4 biological replicates. **C.** Correlation scatter plot between *E. faecalis* strain 24 h CFU/mL in co-culture with *S. mutans* UA159 erm^R and *E. faecalis* strain gelatinase activity. Each dot represents the mean of biological replicates for a strain of *E. faecalis. D.* CFU/mL of indicated *S. aureus* strain in 1:10 co-culture with *S. mutans* UA159 erm^R in BHI at 0, 2, 4, 6, and 24 h. For each culture, n = 2 technical replicates for each of n = 4-6 biological replicates. **E.** CFU/mL of indicated *S. aureus* LAC* strains in 1:10 co-culture with *S. mutans* UA159 erm^R in BHI at 0, 2, 4, 6, and 24 h. For each culture, n = 2 technical replicates for each of n = 4 biological replicates. For panels A, B, D, and E, two-way ANOVA was used for statistical analysis (*, p ≤ 0.05; ***, p ≤ 0.001; ****, p ≤ 0.0001; not significant comparisons are not shown; for panel A, significantly higher mean relative to parental OG1RF comparisons are not shown; for panels B, D, and E, significantly higher mean relative to 0 h comparisons are not shown); each dot represents the mean of technical replicates; floating bars represent the minimum, mean, and maximum; dashed lines indicate limit of detection (LOD); data points at y = 0 indicate no CFUs were detected.

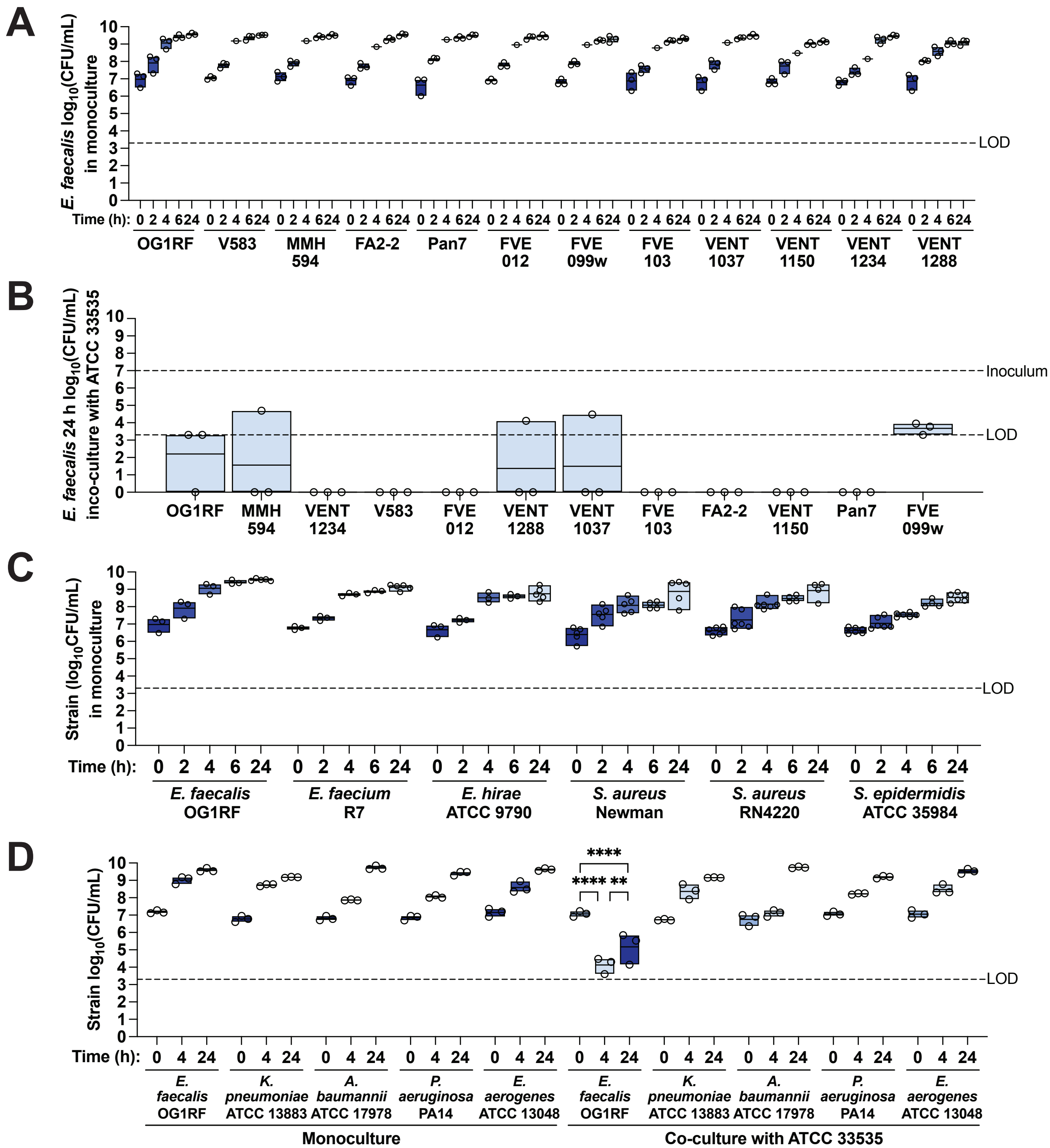

Fig. S4. ***S. mutans*** **ATCC 33535 reduces viability of** ***E. faecalis*** **clinical isolates and** ***Enterococcus*** **spp. strains.**
A. CFU/mL of indicated *E. faecalis* clinical isolate monoculture in BHI at 0, 2, 4, 6, and 24 h performed in parallel with the co-culture experiment shown in Fig. 3B. For each culture, n = 2 technical replicates for each of n = 1-3 biological replicates. **B.** CFU/mL of indicated *E. faecalis* clinical isolates in 1:10 co-culture with *S. mutans* ATCC 33535 in BHI at 24 h (data are the same as shown in Fig. 3A). For each co-culture, n = 2 technical replicates for each of n = 3 biological replicates. Data points at y = 0 indicate no CFUs were detected. **C.** CFU/mL of indicated strains in monoculture in BHI at 0, 2, 4, 6, and 24 h performed in parallel with the co-culture experiment shown in Fig. 3C. For each culture, n = 2 technical replicates for each of n = 3-6 biological replicates. **D.** CFU/mL of indicated strains in monoculture and in 1:10 co-culture with *S. mutans* ATCC 33535 in BHI at 0, 4, and 24 h. For each culture, n = 2 technical replicates for each of n = 3 biological replicates. For panels A, C, and D, two-way ANOVA was used for statistical analysis (**, $p \leq 0.01$; ****, $p \leq 0.0001$; not significant and significantly higher mean relative to 0 h comparisons are not shown); each dot represents the mean of technical replicates; floating bars represent the minimum, mean, and maximum; dashed lines indicate limit of detection (LOD).

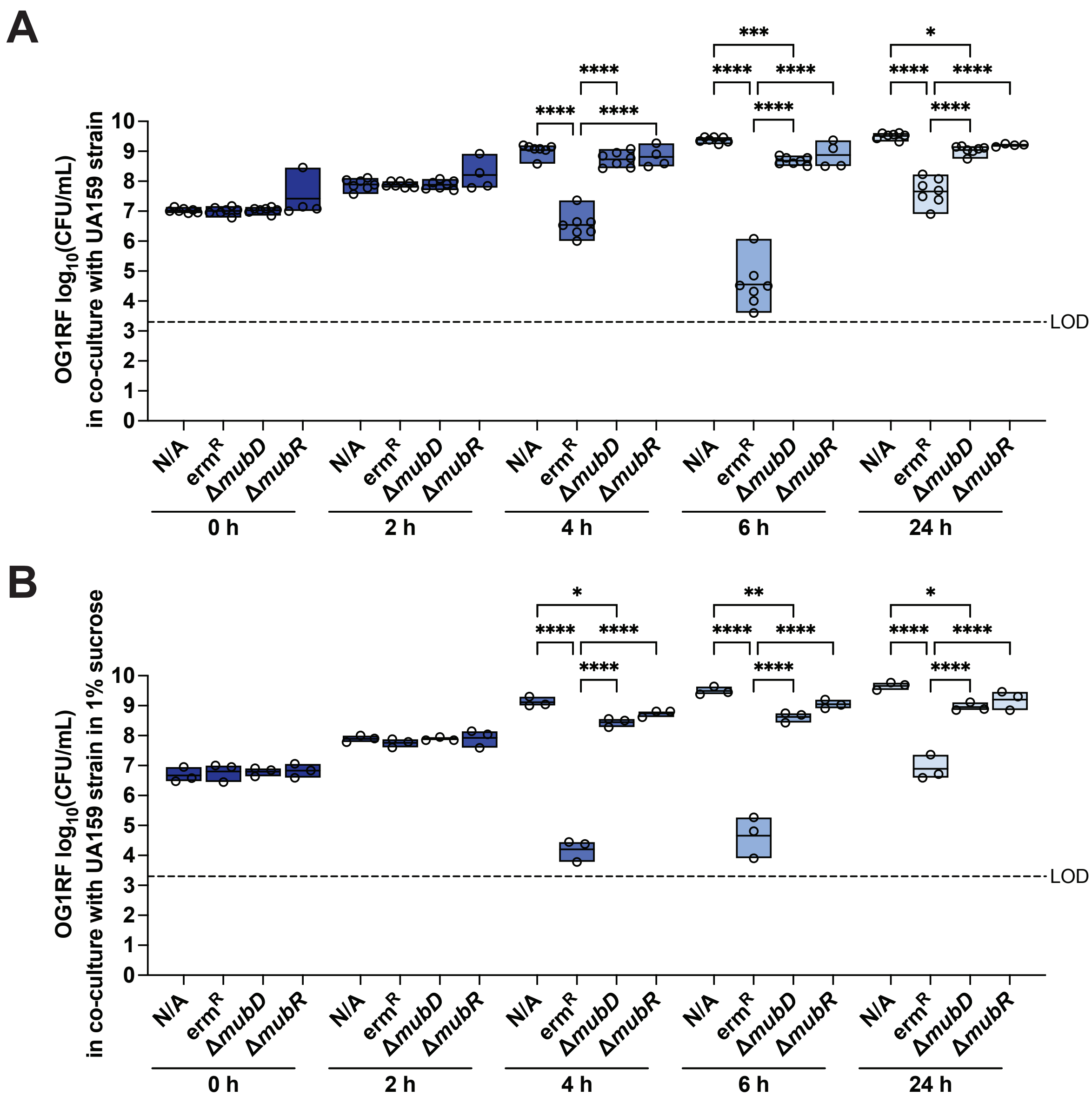

Fig. S5. *S. mutans* UA159 mutanobactin genes are required to reduce viability of *E. faecalis* in co-culture.
A. *E. faecalis* OG1RF CFU/mL in 1:10 co-culture with indicated *S. mutans* UA159 strain in BHI at 0, 2, 4, 6, and 24 h. For each culture, n = 2 technical replicates for each of n = 4-7 biological replicates. **B.** *E. faecalis* OG1RF CFU/mL in 1:10 co-culture with indicated *S. mutans* UA159 strain in BHI + 1% sucrose at 0, 2, 4, 6, and 24 h. For each culture, n = 2 technical replicates for each of n = 3 biological replicates. For panels A and B, two-way ANOVA was used for statistical analysis (*, $p \leq 0.05$; **, $p \leq 0.01$; ****, $p \leq 0.0001$; not significant and significantly higher mean relative to monoculture comparisons are not shown); each dot represents the mean of technical replicates; floating bars represent the minimum, mean, and maximum; dashed lines indicate limit of detection (LOD).

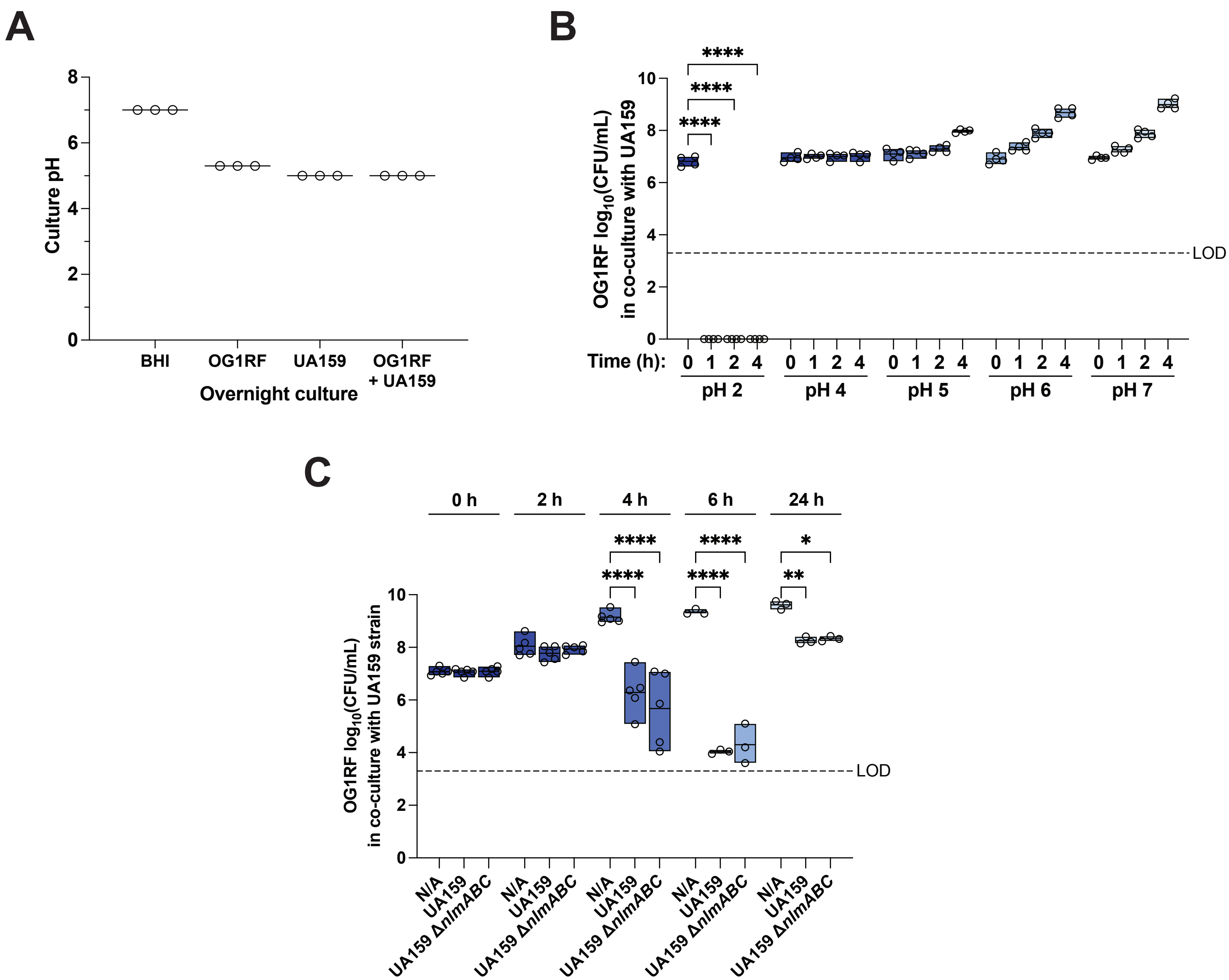

Fig. S6. *E. faecalis* viability is not impacted by culture pH change by *S. mutans* UA159 or by UA159 mutacin production. A. pH of indicated overnight monoculture or co-culture. For each culture, n = 3 biological replicates. Each dot represents each biological replicate. **B.** E. faecalis OG1RF CFU/mL cultured in BHI adjusted to indicated pH at 0, 1, 2, and 4 h. For each culture, n = 2 technical replicates for each of n = 4 biological replicates. **C.** *E. faecalis* OG1RF CFU/mL in co-culture with indicated *S. mutans* UA159 strain in BHI at 0, 2, 4, 6, and 24 h. For each culture, n = 2 technical replicates for each of n = 3-5 biological replicates. For panels B and C, two-way ANOVA was used for statistical analysis (*, $p \leq 0.05$; **, $p \leq 0.01$; ****, $p \leq 0.0001$; not significant comparisons are not shown; for panel B, significantly higher mean relative to 0 h comparisons are not shown; for panel C, significantly higher mean relative to monoculture comparisons are not shown); each dot represents the mean of technical replicates; floating bars represent the minimum, mean, and maximum; dashed lines indicate limit of detection (LOD).

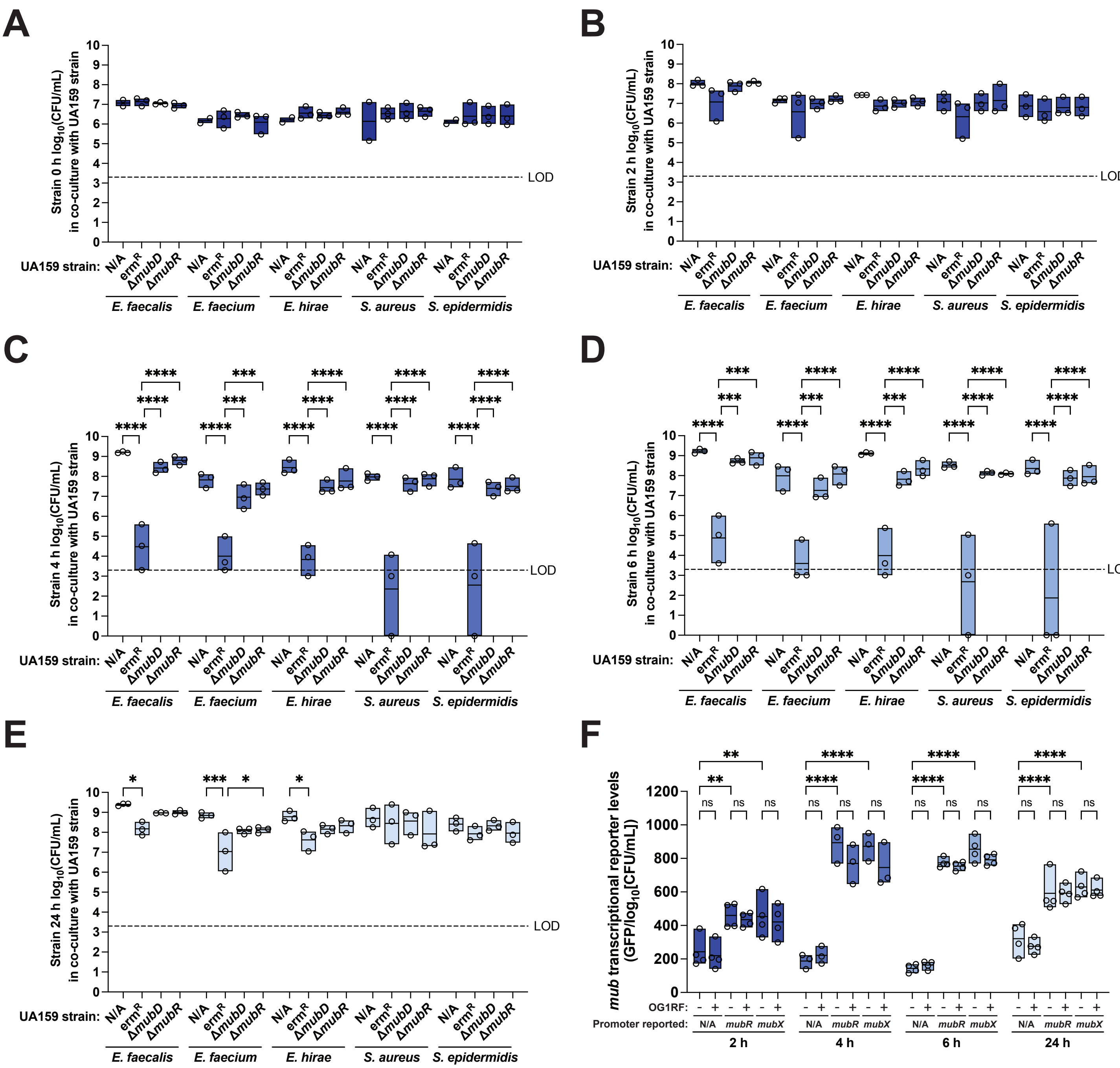

Fig. S7. ***S. mutans*** **UA159 mutanobactin genes are required to reduce viability of target strains in co-culture.**
A. CFU/mL of indicated strain in co-culture with indicated *S. mutans* UA159 strain in BHI at 0 h. For each culture, n = 2 technical replicates for each of n = 2-3 biological replicates. **B.** CFU/mL of indicated strain in co-culture with indicated *S. mutans* UA159 strain in BHI at 2 h. For each culture, n = 2 technical replicates for each of n = 3 biological replicates. **C.** CFU/mL of indicated strain in co-culture with indicated *S. mutans* UA159 strain in BHI at 4 h. For each culture, n = 2 technical replicates for each of n = 3 biological replicates. **D.** CFU/mL of indicated strain in co-culture with indicated *S. mutans* UA159 strain in BHI at 6 h. For each culture, n = 2 technical replicates for each of n = 3 biological replicates. **E.** CFU/mL of indicated strain in co-culture with indicated *S. mutans* UA159 strain in BHI at 24 h. For each culture, n = 2 technical replicates for each of n = 3 biological replicates. **F.** Quantification of *S. mutans* UA159 *mub* transcription in monoculture or in co-culture with *E. faecalis* OG1RF using the pP_{mubR}-*gfp* and pP_{mubX}-*gfp* transcriptional reporter plasmids. For each culture, n = 2 technical replicates for each of n = 3 biological replicates. For panels A-F, two-way ANOVA was used for statistical analysis (*, $p \leq 0.05$; ***, $p \leq 0.001$; ****, $p \leq 0.0001$; for panels A-E, not significant comparisons are not shown); each dot represents the mean of technical replicates; floating bars represent the minimum, mean, and maximum; dashed lines indicate limit of detection (LOD).

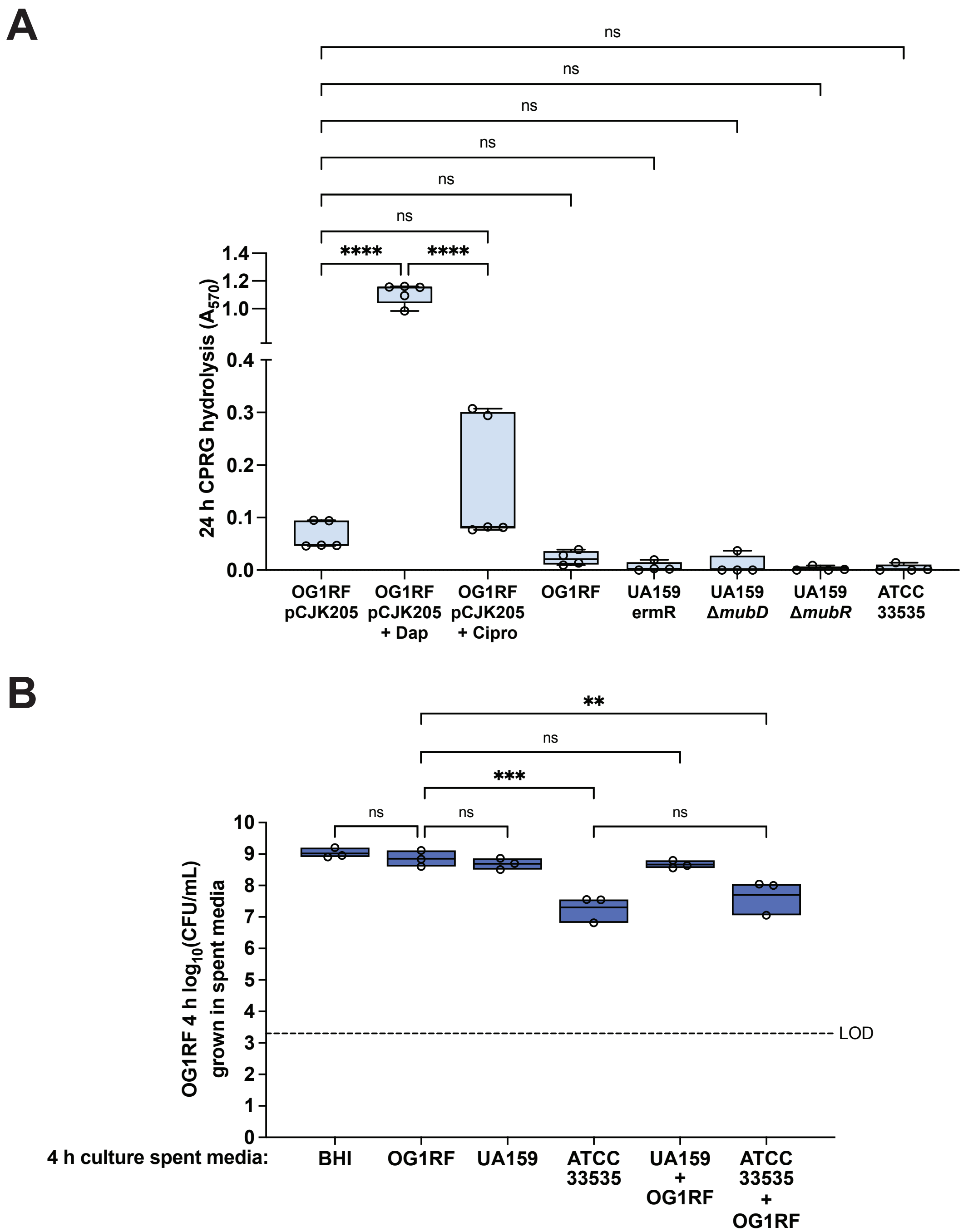

Fig. S8. Strains lacking *lacZ* overexpression plasmid do not significantly contribute to CPRG hydrolysis, *S. mutans* UA159 reduction of *E. faecalis* viability is contact-dependent. A. CPRG hydrolysis of *E. faecalis* OG1RF constitutively expressing *lacZ* from pCJK205 in the presence of subinhibitory concentrations of indicated antibiotic and of *E. faecalis* OG1RF and indicated *S. mutans* strains without pCJK205 at 24 h culture in TBS-D. For each culture, n = 2 technical replicates for each of n = 4-5 biological replicates. **B.** *E. faecalis* OG1RF CFU/mL grown in BHI containing 50% spent media from indicated 4 h monoculture or co-culture. For each culture, n = 2 technical replicates for each of n = 3 biological replicates, dashed line indicates limit of detection (LOD), and data points at y = 0 indicate no CFUs were detected. For panels A and B, one-way ANOVA was used for statistical analysis (ns, not significant; **, $p \leq 0.01$; ***, $p \leq 0.001$; ****, $p \leq 0.0001$); each dot represents the mean of technical replicates; floating bars represent the minimum, mean, and maximum; dashed lines indicate limit of detection (LOD).

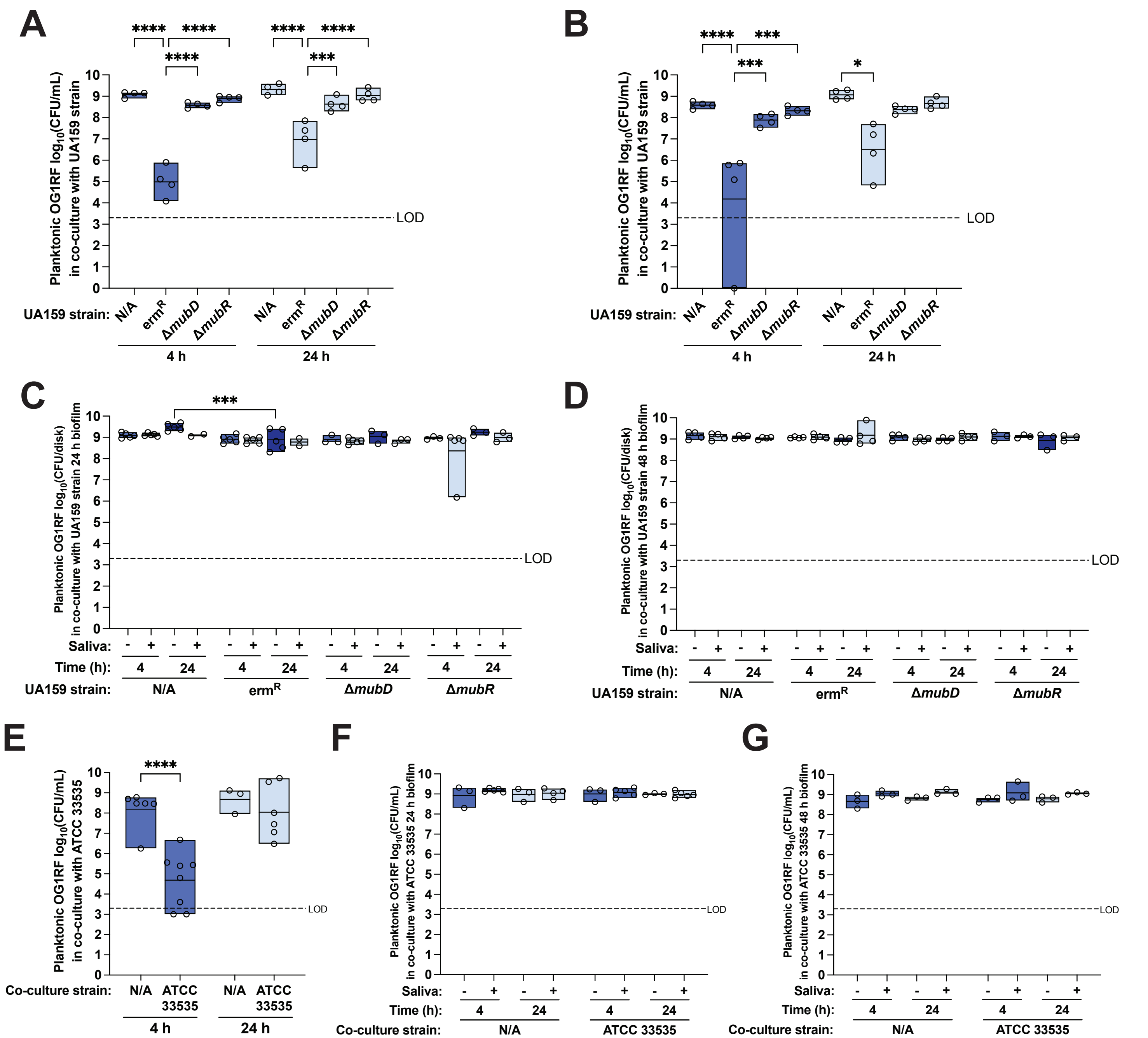

Fig. S9. Planktonic *S. mutans* UA159 and ATCC 33535 reduce viability of *E. faecalis* planktonic cells in the presence of an Aclar disk A. Planktonic *E. faecalis* OG1RF CFU/mL in 1:10 co-culture with indicated *S. mutans* UA159 strain in BHI at 4 and 24 h. For each co-culture, n = 2 technical replicates for each of n = 3-4 biological replicates. **B.** Planktonic *E. faecalis* OG1RF CFU/mL from indicated *S. mutans* UA159 strain in co-culture with OG1RF biofilm pre-formed for 24 h on Aclar in BHI at 4 and 24 h. For each co-culture, n = 2 technical replicates for each of n = 4 biological replicates. **C.** Planktonic *E. faecalis* OG1RF CFU/mL from OG1RF co-cultured with indicated *S. mutans* UA159 strain biofilm pre-formed for 24 h on Aclar ± saliva coating in BHI + 1% sucrose at 4 and 24 h. For each co-culture, n = 2 technical replicates for each of n = 2-5 biological replicates. **D.** Planktonic *E. faecalis* OG1RF CFU/mL from OG1RF co-cultured with indicated *S. mutans* UA159 strain biofilm pre-formed for 48 h on Aclar ± saliva coating in BHI + 1% sucrose at 4 and 24 h. For each co-culture, n = 2 technical replicates for each of n = 3-4 biological replicates. **E.** Planktonic *E. faecalis* OG1RF CFU/mL from *S. mutans* ATCC 33535 in co-culture with OG1RF biofilm pre-formed for 24 h on Aclar in BHI at 4 and 24 h. For each co-culture, n = 2 technical replicates for each of n = 3-8 biological replicates. **F.** Planktonic *E. faecalis* OG1RF CFU/mL from *S. mutans* ATCC 33535 in co-culture with OG1RF biofilm pre-formed for 24 h on Aclar in co-culture in BHI at 4 and 24 h. For each co-culture, n = 2 technical replicates for each of n = 3-5 biological replicates. **G.** Planktonic *E. faecalis* OG1RF CFU/mL from *S. mutans* ATCC 33535 in co-culture with OG1RF biofilm pre-formed for 48 h on Aclar in co-culture in BHI at 4 and 24 h. For each co-culture, n = 2 technical replicates for each of n = 3 biological replicates. For panels A-G, two-way ANOVA was used for statistical analysis (*, $p \leq 0.05$; ***, $p \leq 0.001$; ****, $p \leq 0.0001$; not significant comparisons are not shown); each dot represents the mean of technical replicates; floating bars represent the minimum, mean, and maximum; dashed lines indicate limit of detection (LOD).

Table S1. Strains used in this study

**Strain**	**Description**	**Source or Reference(s)**
S. gordonii		
JW431	DL1	(1)
S. sanguinis		
JW433	SK36	(2)
S. mutans		
JW432	UA159 (Clarke/ATCC 700610; serotype c)	(3)
JW826	UA159B	This study
JW829	UA159 Δ*nlmABC* (Kan^R, Erm^R)	(4)
JW709	UA159 erm^R (*ldh*/*SMU_1116c* IG::*erm*, Erm^R)	This study
JW836	UA159 Δ*mubD*::*erm* (Erm^R)	This study
JW1000	UA159 Δ*mubR*::*erm* (Erm^R)	This study
JW837	UA140 (serotype c)	(5)
JW838	Ingbritt (serotype c)	(6)
JW841	ATCC 33535 (serotype c)	American Type Culture Collection
JW842	ATCC 25175 (serotype c)	American Type Culture Collection
S. sobrinus		
JW840	ATCC 33402 (serotype g)	(7)
E. faecalis		
	OG1RF (Rif^R, Fus^R)	(8)
JW123	OG1RF pJW76 (Tet^R)	(9)
TX5264	OG1RF Δ*gelE* (Tet^R)	(10)
JW659	OG1RF Δ*gelE* pJW76 (Tet^R)	(11)
JW1017	OG1RF Δ*gelE* pJW1016 (Tet^R)	This study
JW329	OG1RF pCJK205 (Erm^R)	(12)
JW620	V583	(13)
	MMH594	(14)
	FA2-2	(15)
	Pan7	(16)
	FVE012	(17)
	FVE099w	(17)
	FVE103	(17)
	VENT1037	(17)
	VENT1150	(17)

	VENT1234	(17)
	VENT1288	(17)
E. faecium		
	R7	(18)
	ATCC 49224	American Type Culture Collection
	ATCC 51558	American Type Culture Collection
	MPH 3-3051	(19)
	MPH 3-1513	(19)
	MPH 4-131	(19)
	MPH 3-1573	(19)
JW728	8E9	(20)
E. hirae		
	ATCC 9790	American Type Culture Collection
S. aureus		
	Newman	(21)
	RN4220	(22)
JW1012	LAC* (USA300, CA-MRSA, erm^S isolate); AH1263	(23)
JW1013	LAC* Δ*aur* Δ*sspAB* Δ*scpA spl::erm* (Erm^R); AH1919	(24)
S. epidermidis		
	ATCC 35984	American Type Culture Collection
K. pneumoniae		
JW1008	ATCC 13883	American Type Culture Collection
A. baumannii		
JW1005	ATCC 17978	American Type Culture Collection
P. aeruginosa		
JW1007	PA14	American Type Culture Collection
E. aerogenes		
JW1006	ATCC 13048	American Type Culture Collection
E. coli		
JW76	DH5α pJW76 (Tet^R)	(9)
JW1016	DH5α pJW1016 (Tet^R)	This study

Table S2. Plasmids used in this study

Plasmid	Description	Source or Reference(s)
pMSP3535	Shuttle vector with nisin-inducible promoter; pAMβ1 and ColE1 replicons, *nisRK*, P*nisA* (Erm^R)	(25)
pJW76	cCF10 pheromone-inducible plasmid for gene expression (Tet^R)	(9)
pJW1016	pJW76 harboring OG1RF *gelE* for cCF10 pheromone-inducible expression (Tet^R)	This study
pCJK205	Constitutive *lacZ* (Erm^R)	(12)
pGFP-Spec	Promoterless pTCV-P23-GFP-Spec (Spec^R)	This study
pJW1204	pGFP-Spec P_{mubR}-*gfp*$^+$	This study
pJW1205	pGFP-Spec P_{mubX}-*gfp*$^+$	This study

Table S3. Primer list

Primer Name	Sequence (5'-3')[a]	Notes
JW288	TACTGCTCGTTTCCGTC	Forward primer to amplify upstream homology region to insert erm^R cassette in the intergenic region between *S. mutans* UA159 *ldh* (SMU_1115) and SMU_1116c genes
JW289	cgctacggatcagatctAACGTTTAATAAAAATACCAAGC	Reverse primer to amplify upstream homology region to insert erm^R cassette in the intergenic region between *S. mutans* UA159 *ldh* (SMU_1115) and SMU_1116c genes
JW290	gcttggtatttttattaaacgttAGATCTGATCCGTAGCG	Forward primer to amplify erm^R cassette from pMSP3535 with homology arms to insert in the intergenic region between *S. mutans* UA159 *ldh* (SMU_1115) and SMU_1116c genes
JW291	gcaggacttactacagtgattaaCTCTTTTTGTGTAATAAAATTCACGC	Reverse primer to amplify erm^R cassette from pMSP3535 with homology arms to insert in the intergenic region between *S. mutans* UA159 *ldh* (SMU_1115) and SMU_1116c genes
JW292	gcgtgaattttattacacaaaaagagTTAATCACTGTAGTAAGTCCTGC	Forward primer to amplify downstream homology region to insert erm^R cassette in the intergenic region between *S. mutans* UA159 *ldh* (SMU_1115) and SMU_1116c genes
JW293	TCTGATCGTAGTCAAAGCC	Reverse primer to amplify downstream homology region to insert erm^R cassette in the intergenic region between *S. mutans* UA159 *ldh* (SMU_1115) and SMU_1116c genes

JW386	ataGGATCCaggaaactggggaaggag	Forward primer to amplify *E. faecalis* OG1RF *gelE* + RBS with BamHI restriction site to clone into pJW76
RYI011	cgatGCTAGCtcattcattgaccagaacagattc	Reverse primer to amplify *E. faecalis* OG1RF *gelE* + RBS from OG1RF with NheI restriction site to clone into pJW76
JW50	ACTCTTCTACAGAAATCGGTC	Forward primer flanking pJW76 MCS to confirm gene insertion
JW199	TCACGAACGAAAATCAAGCC	Forward primer flanking pJW76 MCS to confirm gene insertion
JW365	TCGTACTGAAAGGATCAATATGG	Forward primer to amplify upstream homology region to replace *S. mutans* UA159 *mubD* (SMU_1339) with erm^R cassette
JW366	TATAATCCCTTTCTTCATTCAAAGG	Reverse primer to amplify upstream homology region to replace *S. mutans* UA159 *mubD* (SMU_1339) with erm^R cassette
JW367	gaaatgaagaaagggattataAGATCTG ATCCGTAGCGG	Forward primer to amplify erm^R cassette from pMSP3535 to replace *S. mutans* UA159 *mubD* (SMU_1339)
JW368	cgatatcacctttTCTTTTTTAAAACATTTATTT CCTCCCGTTAAATAATAG	Reverse primer to amplify erm^R cassette from pMSP3535 to replace *S. mutans* UA159 *mubD* (SMU_1339)
JW369	AAAAAGAAAAGGTGATATCGTG	Forward primer to amplify downstream homology region to replace *S. mutans* UA159 *mubD* (SMU_1339) with erm^R cassette
JW370	AGCAATGAACACATAAGCC	Reverse primer to amplify downstream homology region to replace *S. mutans* UA159 *mubD* (SMU_1339) with erm^R cassette
JW371	TCCGCTTCGAACATGAATG	Forward primer to amplify upstream homology region to replace *S. mutans* UA159 *mubR* (SMU_1349) with erm^R cassette
JW372	AAAACCTTCCCTCTCCTTAC	Reverse primer to amplify upstream homology region to replace *S. mutans* UA159 *mubR* (SMU_1349) with erm^R cassette
JW373	gtaaggagagggaaggttttAGATCTGATCCGT AGCGG	Forward primer to amplify erm^R cassette from pMSP3535 to replace *S. mutans* UA159 *mubR* (SMU_1349)
JW374	gagctttgtcttcagtctaaTCTTTTTGTGTAATA AAATTCACGC	Reverse primer to amplify erm^R cassette from pMSP3535 to replace *S. mutans* UA159 *mubR* (SMU_1349)
JW375	TTAGACTGAAGACAAAGCTC	Forward primer to amplify downstream homology region to replace *S. mutans* UA159 *mubR* (SMU_1349) with erm^R cassette

JW376	TGGATGCAAAATGCGC	Reverse primer to amplify downstream homology region to replace *S. mutans* UA159 *mubR* (SMU_1349) with erm^R cassette
RYI009	GAATCATTCATCTTATAATGATGCGCCT	Forward primer flanking *S. mutans* UA159 *mubR* to confirm deletion
RYI010	ACTTTGTACTGGATACGCTCAATCA	Reverse primer flanking *S. mutans* UA159 *mubR* to confirm deletion
RYI018	ATGGTTTCTAAAGGTGAAGAATTGTTTACA	Forward primer to linearize and amplify pGFP_Spec for Gibson assembly to insert promoter of interest directly upstream of GFP ORF
RYI019	ATGTTTTTCCTCCTTATAAAGTGGATCCC	Reverse primer to linearize and amplify pGFP_Spec for Gibson assembly to insert promoter of interest directly upstream of GFP ORF
RYI020	AAGAGAAGTCTGTAAGTTTTGGTTGAG	Forward primer 500 bp upstream of GFP start codon in pGFP-Spec to confirm promoter insertion
RYI021	GAACAGAACCATCTTCAATATTATGACGA	Forward primer 532 bp downstream of GFP start codon in pGFP-Spec to confirm promoter insertion
RYI022	tttataaggaggaaaaacatTCCGATTAGGTCTAAATTCATTATG	Forward primer to amplify 345 bp upstream of UA159 mubR ORF for Gibson assembly into pP23-GFP_Spec upstream of GFP ORF to construct P_{mubR}-GFP reporter plasmid
RYI023	tcttcacctttagaaaccatAAAACCTTCCCTCTCCTTACTTAGTAG	Reverse primer to amplify 345 bp upstream of UA159 *mubR* ORF for Gibson assembly into pP23GFP_Spec upstream of GFP ORF to construct P_{mubR}-GFP reporter plasmid
RYI024	tttataaggaggaaaaacatAAAACCTTCCCTCTCCTTACTTAGTAG	Forward primer to amplify 352 bp upstream of UA159 *mubX* ORF for Gibson assembly into pP23-GFP_Spec upstream of GFP ORF to construct P_{mubR}-GFP reporter plasmid
RYI025	tcttcacctttagaaaccatCTTTTCCTCCGATTAGGTCTAAATTCATT	Reverse primer to amplify 352 bp upstream of UA159 mubX ORF for Gibson assembly into pGFP_Spec upstream of GFP ORF to construct P_{mubR}-GFP reporter plasmid

[a]5'-end lowercase sequences are not complementary to amplified templates. For JW386 and RYI011, uppercase indicates endonuclease recognition sites.

REFERENCES

1. Cisar JO, Kolenbrander PE, McIntire FC. 1979. Specificity of coaggregation reactions between human oral streptococci and strains of *Actinomyces viscosus* or *Actinomyces naeslundii*. Infect Immun 24:742–752.

2. Kilian M, Mikkelsen L, Henrichsen J. 1989. Taxonomic Study of Viridans Streptococci: Description of *Streptococcus gordonii* sp. nov. and Emended Descriptions of *Streptococcus sanguis* (White and Niven 1946), *Streptococcus oralis* (Bridge and Sneath 1982), and *Streptococcus mitis* (Andrewes and Horder 1906). Int J Syst Bacteriol 39:471–484.

3. Ajdić D, McShan WM, McLaughlin RE, Savić G, Chang J, Carson MB, Primeaux C, Tian R, Kenton S, Jia H, Lin S, Qian Y, Li S, Zhu H, Najar F, Lai H, White J, Roe BA, Ferretti JJ. 2002. Genome sequence of *Streptococcus mutans* UA159, a cariogenic dental pathogen. Proc Natl Acad Sci U S A 99:14434–14439.

4. Hossain MS, Biswas I. 2011. Mutacins from *Streptococcus mutans* UA159 are active against multiple streptococcal species. Appl Environ Microbiol 77:2428–2434.

5. Qi F, Chen P, Caufield PW. 2001. The group I strain of *Streptococcus mutans*, UA140, produces both the lantibiotic mutacin I and a nonlantibiotic bacteriocin, mutacin IV. Appl Environ Microbiol 67:15–21.

6. Markham JL, Knox KW, Wicken AJ, Hewett MJ. 1975. Formation of extracellular lipoteichoic acid by oral streptococci and lactobacilli. Infect Immun 12:378–386.

7. Sales MJ, Herbert WG, Du Y, Sandur AS, Stanley NM, Jensen PA. 2018. Complete genome sequences of *Streptococcus sobrinus* SL1 (ATCC 33478 = DSM 20742), NIDR 6715-7 (ATCC 27351), NIDR 6715-15 (ATCC 27352), and NCTC 10919 (ATCC 33402). Microbiol Resour Announc 7:e00804–18.

8. Dunny GM, Brown BL, Clewell DB. 1978. Induced cell aggregation and mating in *Streptococcus faecalis*: evidence for a bacterial sex pheromone. Proc Natl Acad Sci U S A 75:3479–3483.

9. Willett JLE, Ji MM, Dunny GM. 2019. Exploiting biofilm phenotypes for functional characterization of hypothetical genes in *Enterococcus faecalis*. NPJ Biofilms Microbiomes 5:23.

10. Sifri CD, Mylonakis E, Singh KV, Qin X, Garsin DA, Murray BE, Ausubel FM, Calderwood SB. 2002. Virulence Effect of *Enterococcus faecalis* Protease Genes and the Quorum-Sensing Locus *fsr* in *Caenorhabditis elegans* and Mice. Infection and Immunity 70:5647–5650.

11. Willett JLE, Robertson EB, Dunny GM. 2022. The phosphatase Bph and peptidyl-prolyl isomerase PrsA are required for gelatinase expression and activity in *Enterococcus faecalis*. J Bacteriol 204:e0012922.

12. Djorić D, Kristich CJ. 2015. Oxidative stress enhances cephalosporin resistance of *Enterococcus faecalis* through activation of a two-component signaling system. Antimicrob Agents Chemother 59:159–169.

13. Sahm DF, Kissinger J, Gilmore MS, Murray PR, Mulder R, Solliday J, Clarke B. 1989. *In vitro* susceptibility studies of vancomycin-resistant *Enterococcus faecalis*. Antimicrobial agents and chemotherapy 33:1588–1591.

14. Huycke MM, Spiegel CA, Gilmore MS. 1991. Bacteremia caused by hemolytic, high-level gentamicin-resistant *Enterococcus faecalis*. Antimicrob Agents Chemother 35:1626–1634.

15. Clewell DB, Tomich PK, Gawron-Burke MC, Franke AE, Yagi Y, An FY. 1982. Mapping of *Streptococcus faecalis* plasmids pAD1 and pAD2 and studies relating to transposition of Tn917. J Bacteriol 152:1220–1230.

16. Gilmore MS, Rauch M, Ramsey MM, Himes PR, Varahan S, Manson JM, Lebreton F, Hancock LE. 2015. Pheromone killing of multidrug-resistant *Enterococcus faecalis* V583 by native commensal strains. Proc Natl Acad Sci U S A 112:7273–7278.

17. Leuck A-M, Johnson JR, Dunny GM. 2014. A widely used *in vitro* biofilm assay has questionable clinical significance for enterococcal endocarditis. PLoS One 9:e107282.

18. Heaton MP, Handwerger S. 1995. Conjugative mobilization of a vancomycin resistance plasmid by a putative *Enterococcus faecium* sex pheromone response plasmid. Microb Drug Resist 1:177–183.

19. Kristich CJ, Manias DA, Dunny GM. 2005. Development of a method for markerless genetic exchange in *Enterococcus faecalis* and its use in construction of a *srtA* mutant. Appl Environ Microbiol 71:5837–5849.

20. Geldart K, Kaznessis YN. 2017. Characterization of Class IIa Bacteriocin Resistance in *Enterococcus faecium*. Antimicrob Agents Chemother 61:e02033–16.

21. Pollitt EJG, Szkuta PT, Burns N, Foster SJ. 2018. *Staphylococcus aureus* infection dynamics. PLoS Pathog 14:e1007112.

22. Kreiswirth BN, Löfdahl S, Betley MJ, O'Reilly M, Schlievert PM, Bergdoll MS, Novick RP. 1983. The toxic shock syndrome exotoxin structural gene is not detectably transmitted by a prophage. Nature 305:709–712.

23. Boles BR, Thoendel M, Roth AJ, Horswill AR. 2010. Identification of genes involved in polysaccharide-independent *Staphylococcus aureus* biofilm formation. PLoS One 5:e10146.

24. Wörmann ME, Reichmann NT, Malone CL, Horswill AR, Gründling A. 2011. Proteolytic cleavage inactivates the *Staphylococcus aureus* lipoteichoic acid synthase. J Bacteriol 193:5279–5291.

25. Bryan EM, Bae T, Kleerebezem M, Dunny GM. 2000. Improved vectors for nisin-controlled expression in gram-positive bacteria. Plasmid 44:183–190.
